# Efficacy and safety of pertussis vaccination for pregnant women – a systematic review of randomised controlled trials and observational studies

**DOI:** 10.1186/s12884-017-1559-2

**Published:** 2017-11-22

**Authors:** Marie Furuta, Jacqueline Sin, Edmond S. W. Ng, Kay Wang

**Affiliations:** 1 Department of Human Health Sciences, Kyoto University, Graduate School of Medicine, 53 Kawara-cho Shogo-in, Sakyo-ku, Kyoto, 606-8507 Japan; 2 University of Reading, School of Psychology & Clinical Language Sciences, Earley Gate, Reading, RG6 6AL UK; 3 Director’s Office, London School of Hygiene & Tropical Medicine, Keppel St, London, WC1E 7HT UK; 4 Nuffield Department of Primary Care Health Sciences, Radcliffe Primary Care, Radcliffe Observatory Quarter, University of Oxford, Woodstock Road, Oxford, OX2 6GG UK

## Abstract

**Background:**

Worldwide, pertussis remains a major health problem among children. During the recent outbreaks of pertussis, maternal antenatal immunisation was introduced in several industrial countries. This systematic review aimed to synthesize evidence for the efficacy and safety of the pertussis vaccination that was given to pregnant women to protect infants from pertussis infection.

**Methods:**

We searched literature in the Cochrane Central Register of Controlled Trials, Medline, Embase, and OpenGrey between inception of the various databases and 16 May 2016. The search terms included ‘pertussis’, ‘whooping cough’, ‘pertussis vaccine,’ ‘tetanus, diphtheria and pertussis vaccines’ and ‘pregnancy’ and ‘perinatal’.

**Results:**

We included 15 articles in this review, which represented 12 study populations, involving a total of 203,835 mother-infant pairs from the US, the UK, Belgium, Israel, and Vietnam. Of the included studies, there were two randomised controlled trials (RCTs) and the rest were observational studies. Existing evidence suggests that vaccinations administered during 19–37 weeks of gestation are associated with significantly increased antibody levels in the blood of both mothers and their newborns at birth compared to placebo or no vaccination. However, there is a lack of robust evidence to suggest whether these increased antibodies can also reduce the incidence of pertussis (one RCT, *n* = 48, no incidence in either group) and pertussis-related severe complications (one observational study) or mortality (no study) in infants. Meanwhile, there is no evidence of increased risk of serious complications such as stillbirth (e.g. one RCT, *n* = 103, RR = 0, meaning no case in the vaccine group), or preterm birth (two RCTs, *n* = 151, RR = 0.86, 95%CI: 0.14–5.21) related to administration of the vaccine during pregnancy.

**Conclusion:**

Given that pertussis infection is increasing in many countries and that newborn babies are at greatest risk of developing severe complications from pertussis, maternal vaccination in the later stages of pregnancy should continue to be supported while further research should fill knowledge gaps and strengthen evidence of its efficacy and safety.

**Electronic supplementary material:**

The online version of this article (10.1186/s12884-017-1559-2) contains supplementary material, which is available to authorized users.

## Background

Worldwide, pertussis remains a major health problem among children [1]. Despite infant vaccination programmes with high coverage rates, many industrialized countries have recently experienced pertussis outbreaks. This is partially due to waning immunity following vaccination, which typically occurs 4–12 years after the last booster dose or episode of illness, as well as to decreases in natural immunity [1, 2]. Pertussis in adults can be a source of infection for infants under 2 months of age who have not yet been vaccinated. These first few months are the period when infants are at greatest risk of developing pertussis infection and serious complications such as pneumonia, seizures or brain damage, any of which could ultimately be fatal [2, 3].

In seeking potential strategies for preventing pertussis in infants, vaccinating babies at birth was not considered as the best protection against pertussis. This is because newborns’ immune systems cannot create antibodies until they are 2 months old [3], so vaccination cannot immediately protect them [4]. Instead, maternal immunisation is considered the only available option for protecting infants against pertussis from birth until the first vaccinations are given at 2 months [1]. Vaccinating women in pregnancy is believed to protect infants from pertussis in two ways. First, it is likely to increase the transfer of maternal pertussis antibodies through the placenta [2, 5] and through breast milk [6]. Second, vaccination during pregnancy is likely to prevent maternal infection at the time of delivery, which, in turn, minimises the infant’s potential exposure to pertussis [1].

During the 2011 outbreaks, the United States (US) became the first country to recommend that health care personnel administer pertussis-containing vaccines to pregnant women who were between 27 and 36 weeks of gestation [7]. Pertussis vaccination during pregnancy is now recommended in several countries, including the United Kingdom (UK), New Zealand, Belgium, Brazil, Argentina, Mexico and Israel, to protect infants from the infection. The rate of pertussis immunisation during pregnancy, however, varies across and within countries. In the UK, approximately 60% of pregnant women received a pertussis vaccine in 2014 [8]. A US study reported that nearly 50% of women (*n* = 5992/12,089) who delivered in Wisconsin from November 2013 to March 2014 received a pertussis-containing vaccine (tetanus-diphtheria-acellular pertussis: Tdap) during pregnancy [9]. There are several factors influencing on uptake of maternal pertussis vaccination such as maternal level of education, maternal work situation and parity [10]. Studies suggest that one of the main reasons for low immunisation rates is clinicians’ and/or women’s concerns about the safety of the vaccine during pregnancy [11–15].

Although increasing evidence suggests that receiving the pertussis vaccine during pregnancy is effective [16] and safe [17, 18], little is known regarding the quality of the evidence. There is also a concern that maternal vaccine-induced antibodies transferred to the fetus may inhibit the infant’s immune response to vaccination [19]. Therefore, the current systematic review aimed to synthesise evidence for the efficacy and safety of pertussis vaccinations that were given to pregnant women to protect their infants from pertussis. Our primary objective was to examine the efficacy of maternal pertussis vaccination in reducing the incidence of infant pertussis compared to placebo or no vaccination. Secondary objectives were to examine the efficacy of maternal pertussis vaccination in: 1) reducing the incidence of severe complications attributed to pertussis in infants (as measured by hospital admissions and/or incidence of pneumonia, seizures, brain damage, or death attributed to pertussis); and 2) increasing infants’ and mothers’ immune responses (as measured by anti-pertussis antibodies in blood or breastmilk). We also examined 3) the safety of maternal pertussis vaccination as measured by adverse vaccine-related outcomes in mothers, and/or incidence of obstetric or perinatal complications, with a focus on clinically important outcomes such as hypertensive disorder (e.g. pre-eclampsia and eclampsia), chorioamnionitis, preterm birth, small-for-gestational-age, stillbirth and neonatal death.

## Methods

### Data sources and searches

We carried out systematic electronic searches in the Cochrane Central Register of Controlled Trials (CENTRAL), Medline, Embase and OpenGrey using a search strategy that was developed in consultation with an information specialist. We used both index terms (e.g. Medical Subject Heading: MeSH) and free-text terms to maximise the search’s sensitivity. The search terms included ‘pertussis’, ‘whooping cough’, ‘pertussis vaccine,’ ‘tetanus, diphtheria and pertussis vaccines’, ‘Tdap’, ‘pregnancy’ and ‘perinatal’ (details are shown in Additional file 1). The citations we retrieved from the searches were imported into the reference management software package EndNote X7. After we removed the duplicates, two authors (MF, JS) independently screened for relevant articles based on titles, abstracts and descriptors/MeSH terms. We retrieved full texts for potentially relevant studies, and two authors (MF, JS) reviewed these articles independently to determine whether they met the inclusion criteria. At the end of each stage, the review authors discussed their screening results and any discrepancies were resolved through consensus. Additional searches were undertaken by hand searching the reference lists of the included studies. The searches were initially completed at the end of February 2015 and updated on 16 May 2016. We recorded the study selection process in detail so as to complete a Preferred Reporting Items for Systematic Reviews and Meta-Analyses (PRISMA) flow diagram.

### Inclusion and exclusion criteria

To be eligible, studies had to compare at least one of the following efficacy or safety outcome measures between two groups: pertussis or pertussis-containing vaccine vs. placebo or no vaccination during pregnancy.

#### Efficacy

- Pertussis infection (either laboratory confirmed or clinically diagnosed) in infants up to 12 months of age (primary outcome)
- Severe complications attributed to pertussis (as measured by hospital admissions and/or incidence of pneumonia, seizures, brain damage or death attributed to pertussis) in infants up to 12 months of age
- Mothers’ and infants’ immune responses (as measured by anti-pertussis antibodies, i.e. pertussis toxin, pertactin, filamentous hemagglutinin and/or fimbriae) in maternal and infant blood at delivery; anti-pertussis antibodies in infant blood prior to the first dose of a pertussis vaccine [at approximately 2 months of age] but after the primary infant vaccination schedule was complete [five to 12 months of age]; or antibodies in breast milk in colostrum and up to 12 months after giving birth)

#### Safety

- Adverse vaccine-related outcomes (e.g. incidence of any solicited adverse events including local and systemic reactions) following injections
- Obstetric or perinatal complications

We included randomised control trials (RCTs) in which individual pregnant women were randomly assigned to a vaccination or a no-vaccination (placebo or no vaccination) group so that differences in outcomes of babies born to women with or without the pertussis vaccination could be examined. If a RCT adopted a crossover design, we only included outcome data from the first randomisation period. We excluded cluster-randomised trials because the purpose of our review was to examine the vaccine’s efficacy and safety at the individual level. Apart from RCTs, we also included quasi-RCTs and observational studies that evaluated the vaccine’s efficacy and safety. We did not apply restrictions based on study settings, but we included only studies written in English.

### Data extraction and risk of bias assessment

Using data extraction forms designed specifically for this review, MF extracted information on study design, participants, interventions or exposures, and outcomes measured. JS double-checked the accuracy of data extraction. Two authors (MF, JS) independently assessed the risk of bias in the included studies, using the Cochrane collaboration risk of bias tool for RCTs, cohort studies and case-control studies [20, 21] as appropriate. We assessed each domain of bias (e.g. sequence generation, allocation concealment, blinding, incomplete outcome, selective reporting for RCTs) and assigned it one of the following three judgments: low risk of bias (plausible bias that was unlikely to seriously alter the results), high risk of bias (plausible bias that seriously weakened confidence in the results), or unclear risk of bias (plausible bias that could have raised some doubt about the results) when there was insufficient information to make a judgment. We used the Grading of Recommendations Assessment, Development and Evaluation (GRADE) approach [22] to assign the overall quality of evidence for each outcome to one of four levels – high, moderate, low, or very low – according to factors including within-study risk of bias (methodological quality), directness of evidence, heterogeneity, precision of effect estimates and risk of publication bias [21, 22].

### Data synthesis and analysis

The extracted outcome data were further checked by a statistician (ESWN) who conducted statistical analyses. We used the Mantel-Haenszel method to estimate the pooled risk ratios (RRs) with 95% confidence intervals (CIs) for all dichotomous outcomes, except for one case-control study for which we produced odds ratios (ORs), because with this type of study design, the entire at-risk population cannot be defined and, thus, we could not calculate RR. For the continuous outcome data, i.e. anti-pertussis antibodies, underlying data were skewed. We therefore calculated the ratios of the geometric means and 95%CIs where group-specific geometric means and associated CIs were reported for the included studies [23]. Test of no difference (null hypothesis) between two geometric means was performed by t-test on the difference of the log-transformed geometric means.

We performed random effects meta-analyses, where possible, using Review Manager (RevMan) 5.3 to produce the average effect sizes of the interventions across studies. We analysed the data from observational studies separately from the trial data. Clinical heterogeneity (variability in the interventions and controls, participants, settings, and outcomes) and methodological heterogeneity (variability in study design and risk of bias) were assessed within each comparison. We also assessed statistical heterogeneity using I-squared and chi-squared statistics [21], as well as visually inspecting the forest plots. We reported study results separately when there was significant heterogeneity in the studies’ design and findings.

## Results

**Fig. 1:**
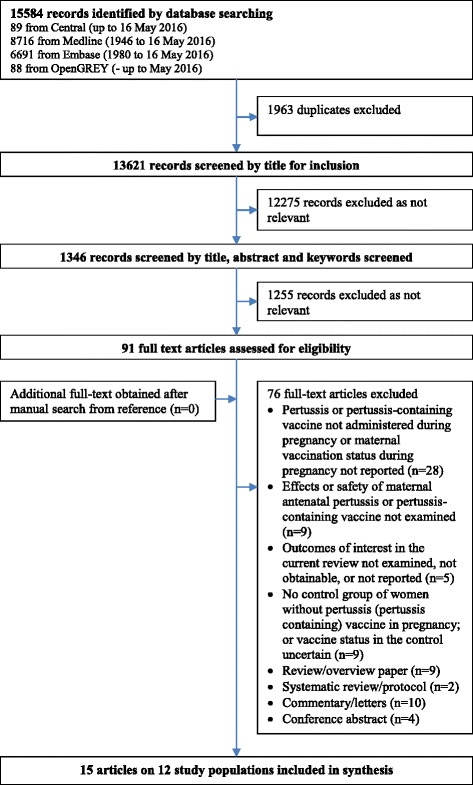
Study selection. Flow diagram of the literature search process and results

Our electronic search identified 15,584 articles. After excluding duplicates, we screened 13,621 titles and/or abstracts. Of potentially relevant articles (*n* = 1347), we examined 91 full-text articles and finally, a total of 15 articles met our inclusion criteria (Fig. 1) (details of excluded studies are shown in Additional file 2). Table 1 summarises the characteristics of included studies. The articles by Kharbanda et al. [24, 25] reported different outcomes in the same study population: one focused on obstetric and perinatal complications [24] and the other focused on adverse events in mothers occurring within 42 days of vaccination [25]. The articles by Abu Raya et al. [26, 27] also reported different outcomes in the same population: one focused on antibodies in maternal and infant blood [26] and the other focused on antibodies in breast milk [27]. In addition, it appears that the eligible sample we included from De Schutter et al. [28] (*n* = 19 women vaccinated during pregnancy; *n* = 9 women with no vaccination for at least 5 years before delivery) were drawn from the participants in Maertens et al. [29]’s study. These studies reported different outcomes: one focused on antibodies in maternal and infant blood as well as obstetric complications [29] and one focused on antibodies in breast milk [28]. Altogether, this represented data from 12 study populations involving a total of 203,835 mother-infant pairs from the US, the UK, Belgium, Israel and Vietnam. In terms of study designs, there were two RCTs (Hoang et al. [30], Munoz et al. [31]), one of which [31] employed a crossover design (women who received saline during pregnancy were given the Tdap postpartum, and women who received it during pregnancy were given saline postpartum). The remaining 10 studies (13 articles) were observational studies: nine cohort studies [5, 24–29, 32–36] and one case-control study [37].

**Table 1:** Characteristics of included studies

Authors	Country	Setting	Study design	Dates of recruitment	Participants Inclusion (I)/Exclusion (E)	Intervention Exposure	Comparisons	N	Outcomes
Abu Raya [26]	Israel	Hospital In−/outpatients not specified	PCS	2013–2014	I: pregnant women with singleton births at gestational age ≥ 36 weeks E: pregnant women with a newborn <2000 g; immunologic disorder; immunoglobulins in the previous year; immunosuppressive drugs in the current pregnancy; pertussis infection or pertussis-containing vaccine within 5 years or any vaccine besides Tdap within 2 weeks of delivery.	Tdap ≥20 weeks’ gestation	No Tdap during pregnancy	81	Anti-pertussis antibodies in mothers’ and infants’ sera measured by ELISA
Abu Raya [27]	Israel	Same as above	PCS	2013–2014	I: pregnant women with singleton births; gestational age ≥ 36 weeks; intending to breast feedE: same as Abu Raya et al. [26]	Tdap ≥20 weeks’ gestation	No Tdap during pregnancy	37	Anti-pertussis antibodies in breast milk measured by ELISA
Dabrera [37]	UK	Community- based data	CCS	2012–2013	E: any potentially confounding protective effect from active immunisation.	Any pertussis-containing vaccine at any time in pregnancy	No pertussis-containing vaccine during pregnancy	113	Pertussis infection in infants; pertussis related complications as measured by the length of hospital stay in infants
De Schutter [28]	Belgium	Hospitals In−/outpatients not specified	PCS	2013–2014	E: Women who delivered prematurely, who had received another vaccine or any blood product in the previous month.	Tdap during the 2nd or 3rd trimester	No Tdap during pregnancy^a^	28^a^	Anti-pertussis antibodies in breast milk measured by ELISA
Donegan [32]	UK	Community- based data	RCS	2012–2013 for vaccine group/ 2010–2012 for control group	I: women aged ≥12 who had a pertussis-containing vaccination during pregnancy and at least 28 days of follow-up data after vaccination	Any pertussis vaccine at any time during pregnancy	No pertussis-containing vaccine during pregnancy	38,900	Obstetric and perinatal complications
Gall [5]	US	Hospital In−/outpatients not specified	RCS	2008–2009	I: pregnant women who had been admitted to the hospital at the study site and their babies	Tdap at any time during pregnancy^b^	No Tdap during pregnancy	104	Anti-pertussis antibodies in infants’ sera measured by ELISA
Hardy-Fairbanks [33]	Not specified^c^	Not specified^b^	PCS	2006 for vaccine group/ 2008–2009 for control group	E: Women with multiple gestations; serious underlying health issues in either the mother or infant, preterm infants, or infants who needed transfusions or who had been advised not to have blood drawn for health reasons	Tdap at any time during pregnancy	No Tdap during pregnancy	70	Anti-pertussis antibodies in mothers’ and infants’ sera measured by ELISA
Healy [34]	US	Hospital In−/outpatients not specified	RCS	2009–2011	I: Women who delivered at ≥37 weeks’ gestation; received Tdap within 2 years; and had plasma–serum pairs available	Tdap at any time during pregnancy	Tdap before pregnancy	105	Anti-pertussis antibodies in mothers’ and infants’ sera measured by ELISA
Hoang [30]	Vietnam	Community	RCT	2012–2013	I: Women aged 18–41 with low risk for complications.E: Women with: any serious underlying medical condition; febrile illness in the 72 h before injection, receipt of TT vaccine in the past month; receipt of Tdap in the past 10 years; receipt of a vaccine, blood product or experimental medicine in the 4 weeks before or after injection; previous severe reaction to any vaccine	Tdap, 20–30 weeks’ gestation	TT during pregnancy	103	Anti-pertussis antibodies in mothers’ and infants’ sera measured by ELISA; vaccine-related adverse outcomes; obstetric and perinatal complications.
Kharbanda [24]	US	Community- based data	RCS	2010–2012	I: Women with singleton pregnancies with a live birth E: women with live virus vaccines during pregnancy; Tdap in the 7 days after the estimated pregnancy start date or in the 7 days before delivery; incomplete birth data	Tdap at any time during pregnancy^d^	No Tdap during pregnancy	123,494	Obstetric and perinatal complications
Kharbanda [25]	US	Community- based data	RCS	2007–2013	Same as Kharbanda et al. [24]	Tdap at any time during pregnancy^d^	No Tdap during pregnancy	163,138	Obstetric complications occurring within 42 days of vaccination.
Ladhani [35]	UK	Community- based data	RCS	2012–2014 for vaccine group/ 2011–2012 for control group	I: Any infant eligible for the national immunisation program	Tdap-IPV	No pertussis -containing vaccine during pregnancy	387	Antibody responses after primary immunisation in infants’ sera measured by ELISA.
Maertens [29]	Belgium	Hospitals In−/outpatients not specified	PCS	2012–2014	I: Women aged 18–40 with low risk for complications. E: same as Hoang et al. [30]	Tdap at 22–33 weeks’ gestation	No pertussis-containing vaccine during pregnancy	99	Anti-pertussis antibodies in mothers’ and infants’ sera measured by ELISA; obstetric complications
Munoz [31]	US	NIH Vaccine Treatment Evaluation Unit	RCT	2008–2012	I: Women aged 18–45 with no underlying chronic medical conditions, a singleton, uncomplicated pregnancy with normal first- or second-trimester screening test results E: Women who received Tdap or any tetanus-containing vaccine within the prior 2 years	Tdap at 30–32 weeks’ gestation	Placebo	45	Anti-pertussis antibodies in mothers’ and infants’ sera measured by ELISA; vaccine-related adverse outcomes; perinatal complications; pertussis infection in infants
Shakib [36]	US	Healthcare database	RCS	2005–2009	I: Pregnant women 12–45 years of age and their babies E: Women who had documentation of Tdap vaccine within 3 days prior to the delivery outcome	Tdap at any time during pregnancy	No Tdap during pregnancy	690	Pertussis infection in infants; perinatal complications

*RCT* randomised control trial, *PCS* prospective cohort study, *RCS* retrospective cohort study, *CCS* case-control study, *Tdap* a combined tetanus toxoid, reduced diphtheria toxoid, acellular pertussis vaccine, *TdaP/IPV* a combined tetanus, low-dose diphtheria, five-component acellular pertussis, inactivated polio vaccine, *TT* tetanus toxoid vaccine, *ELISA* Enzyme-linked immunosorbent assays

^a^De Schutter et al. compared different maternal pertussis vaccination strategies. This review only included women with Tdap during pregnancy (*n* = 19) and women with no pertussis vaccination for at least for 5 years before delivery (*n* = 9)

^b^Women in the study were encouraged to receive Tdap during the second trimester of pregnancy, but the exact timing of its administration could not be determined

^c^Hardy-Fairbanks et al. did not mention the study setting. The study appears, however, to have been conducted in the United States, according to the available information (the authors’ institutional affiliations and the acknowledgements section). The authors also described cases of pertussis that had been reported in New Hampshire during the period when the study participants were recruited

^d^Tdap vaccine received from 8 days after the last menstrual period to seven to 8 days before delivery

Many studies selected women who were at low-risk of obstetric complications; for example, four studies [28–31, 33] recruited healthy pregnant women with no underlying medical conditions, and four [24–27, 31, 33] recruited only women with singleton pregnancies. Preterm birth was excluded in three cohort studies [26–28, 33, 34]. However, one RCT [31] and two cohort studies [24, 36] specified preterm birth as one of their outcomes of interest. Women with a recent history of vaccination and/or pertussis infection were excluded in some studies to minimise any potential confounding of anti-pertussis antibodies [26–31].

### Intervention/exposure and comparison

In six studies (representing eight articles), the intervention or exposure was immunisation during pregnancy with Tdap vaccines [24, 26–31, 33, 34, 36]. All US studies [24, 31, 33, 34, 36] and a Vietnamese RCT [30] used Tdap, manufactured by Sanofi Pasteur (including Adacel, which contains 2.5 μg of pertussis toxoid), whereas studies in Israel [26, 27] and Belgium [28, 29] used Boostrix (containing 8 mcg of pertussis toxoid), manufactured by Glaxo Smith Kline. In the UK studies, the exposure were a combined tetanus, diphtheria, 5-component acellular pertussis, inactivated polio vaccine (TdaP-IPV, Repevax, Sanofi Pasteur) [35] or ‘any vaccine containing pertussis’ administered during pregnancy [32]. In one UK case-control study [37], the type of pertussis vaccination was not reported. The timing of pertussis vaccination administration varied between and within studies from the first to the third trimesters. The comparator of the Tdap vaccination during pregnancy was a placebo in one RCT [31] and the tetanus toxoid (TT) vaccine in another [30]. For observational studies, women in the comparator-group of one study [34] received the vaccination before pregnancy. Otherwise, the comparator for all the remaining observational studies was no vaccination during pregnancy.

### Risk of bias in included studies

The risk of bias assessment for all included studies is summarised in Additional file 3 and described below.

#### RCT

Both RCTs [30, 31] failed to report the processes used to generate the randomisation sequence and allocation concealment; thus, these studies were assessed as having an unclear risk of selection bias. One RCT [31] blinded investigators and participants but not the vaccine administrator, and no information on blinding was available for the RCT conducted in Vietnam [30]. The trials were, however, assessed as having a low risk of bias for the objectively measured outcomes (i.e. antibodies) and an unclear risk of bias for the self-reported outcomes (i.e. injection site and systemic reactions, for which the knowledge of the vaccine’s or the placebo’s uptake might have affected the way the women or the clinicians reported symptoms). In both trials, the proportion of missing outcome data was small for the outcomes measured during pregnancy and delivery, with a 92%–100% completion rate. However, in the trial in Vietnam that examined anti-pertussis antibodies in infants aged either two or 5 months, losses to follow-up were greater than 20% and 30%, respectively, giving the trial data an unclear risk of bias for incomplete outcome data collection. The risk of reporting bias was also rated as unclear in one trial [31] because some of the vaccine-associated complications (e.g. hypertension, poor feeding) that were planned to be measured at various points as stated in the protocol (clinicaltrials.gov identifier: NCT00707148) were not reported in the paper; in another RCT [30], the authors did not mention the protocol in the article, although we found the plausible study protocol from clinicaltrias.gov (identifier: NCT01698346). At the baseline, no imbalance between the intervention and control groups was observed in either RCT. However, with the small sample sizes in each study, the estimated effect sizes were imprecise, leaving room for potential bias.

#### Cohort studies

All twelve included articles had unclear or high risks of bias across several domains. The main issues across studies were (1) matching or adjusting for prognostic factors, or (2) assessing prognostic factors. Five articles were rated as having a high risk [5, 32, 33, 35, 36] and another seven as having an unclear risk of bias for matching or adjustment for prognostic factors [24–29, 34], mainly because of potential confounders (e.g. recent history of pertussis infection or vaccination, or obstetric characteristics) that could be associated with outcomes were not adjusted for (or not comprehensively adjusted for), and/or data required to assess the comparability between groups were not presented. For eight articles [24, 25, 27–29, 32–34], we were also uncertain regarding the reliability (e.g. accuracy and completeness) of data from which information on potential confounders were obtained.

#### Case-control study

For the only case-control study included [37], there were three domains in which the risk of bias was rated as unclear: case selection (some of the eligible pertussis cases were not included in the analysis), matching of cases and controls (no matched controls were selected for 28 of 58 pertussis cases), and matching/adjusting for prognostic factors (some important potential confounders were not adjusted for, such as gestation at vaccination, breastfeeding status, number of children in households, childcare attendance, and socio-demographic factors).

### Efficacy

#### Pertussis infection

One RCT that involved 48 mother-infant pairs [31] and one retrospective cohort study that involved 690 mother-infant pairs [36] reported incidence of pertussis in infants, but neither identified any pertussis cases in either the Tdap-vaccinated or control groups. In both studies, pertussis incidence was not the primary outcome of interest; hence, it is likely that the sample sizes were not calculated to detect differences in incidence. However, one case-control study [37] showed a significant association between maternal antenatal vaccination and pertussis infection in infants aged less than 8 weeks: the rate of pertussis vaccination during pregnancy was significantly lower in mothers of pertussis-positive infants than in mothers of pertussis-negative infants (OR 0.07 [95% CI: 0.03–0.19] after adjustment for sex, geographic area and birth period). It is worth noting that case-control studies are not designed for determining the incidence of a disease, because the case-control ratios are predetermined by the study design [38]. The quality of the evidence for pertussis incidence was therefore rated as low using the GRADE approach because no direct evidence was available on the incidence in infants.

#### Severe complications attributed to pertussis

No study that examined the efficacy of maternal pertussis vaccination during pregnancy reported on differences in rates of hospitalisation, pneumonia, seizures, brain damage or death attributed to pertussis in infants. One case-control study [37], however, reported that a history of maternal pertussis vaccination during pregnancy was not associated with a significant difference in the length of hospital stay for infants (aged <8 weeks) with pertussis (*n* = 47, median: 4 days in infants of mothers with antenatal pertussis vaccine vs. 3.5 days in infants of mothers without the vaccine, *p* = 0.58). The quality of the evidence for severe complications attributed to pertussis was rated as low using the GRADE criteria because the outcome examined, i.e. the length of hospitalisation, was a proxy measure for the severity of pertussis complications, and also, only one case-control study contributed to the outcome.

#### Mothers’ and infants’ immune response to vaccine

##### Antibodies in maternal and infant blood

Seven studies [5, 26, 29–31, 33, 34] evaluated antibodies in paired maternal and cord blood samples, and one evaluated antibodies in the infant blood samples only. The antibodies were measured by enzyme-linked immunosorbent assay (ELISA) kits from different manufacturers including Euroimmun [26, 30] and Virion\Serion [29], although ELISA kits manufacturers were not described in some studies. However, meta-analyses were not possible because the included studies were different both in design (RCT vs. observational) and comparison group (i.e. placebo, tetanus toxoid, no Tdap during pregnancy or Tdap before pregnancy) or because of insufficient data were provided in the original study (i.e. Hardy-Fairbanks et al. [33] reported neither confidence intervals nor standard deviations of the mean differences).

Table 2 shows the antibody measurements in the mothers and infants. Two RCTs [30, 31] and three cohort studies [26, 29, 33] consistently showed higher antibody concentrations at delivery in women who received Tdap during pregnancy than in their controls (e.g. in one RCT [*n* = 48], antibodies in the maternal blood: 51.0 EU/mL [95% CI: 37.1–70.1] in the Tdap-vaccinated group and 9.1 EU/mL [4.6–17.8] in the placebo group; ratio of geometric means 5.6 [3.0–10.5]; *p* < 0.001) and in their infants at birth (e.g. [31], antibodies in the infant cord blood: 68.8EU/mL [52.1–90.8] in the Tdap-vaccinated group and 14.0 EU/mL [7.3–26.9] in the placebo group; ratio of geometric means 4.9 [2.8–8.7]; p < 0.001). There was, however, one cohort study [34] that reported no significant difference between the groups; in that study, antibody levels in maternal and infant blood at delivery were compared between women who received Tdap during early pregnancy (mean = 9 weeks gestational age) and those who received it before pregnancy (10–24 months prior to the birth of the current infant). Sensitivity analyses that removed three women who had most likely had recent pertussis exposure (immunoglobulin G [IgG] to pertussis toxin of >94 EU/mL in maternal sera) did not change the overall findings; data were also not available for subgroup analysis by gestational age at pertussis vaccination. However, nearly all studies in which the majority of women in the vaccinated group received Tdap in late pregnancy showed significantly higher antibody levels in maternal and infant blood than did the control group (mean gestational age at vaccination: 26 weeks and 29 weeks in [30] and [29], respectively; the means were not available, but all women received Tdap at 30–32 weeks in [31], and the majority (58 of 61) received it after 27 weeks in [26]). Only one study in which women received Tdap in early pregnancy (mean = 9 weeks gestational age) did not find significant differences in the maternal and infant blood antibody levels between groups [34].

**Table 2:** Geometric mean concentrations of pertussis antibodies in maternal and infants’ blood at birth

	Study design	Intervention/ exposuretype, mean gestational week at vaccination (range)	Com-parator	Maternal blood birth					Infant cord blood at birth				
				n: Vaccine/control	Vaccine Geometric mean (95% CI)	Control Geometric mean (95% CI)	Ratio of geometric means (95% CI)	p	n: Vaccine/control	Vaccine Geometric mean (95% CI)	Control Geometric mean (95% CI)	Ratio of geometric means (95% CI)	p
PT (EU or IU/ml)*													
Munoz [31]	RCT	Tdap -- (30–32)	placebo	33/14	51.0 (37.1–70.1)	9.1 (4.6–17.8)	5.6 (3.0–10.5)	<0.001	31/14	68.8 (52.1–90.8)	14.0 (7.3–26.9)	4.9 (2.8–8.7)	<0.001
Hoang [30]	RCT	Tdap 26 (19–35)	TT	51/47	17.3 (13.0–22.0)	5.7 (4.3–7.6)	3.0 (2.1–4.4)	<0.001	50/47	21.0 (16.0–28.0)	7.2 (5.6–9.4)	2.9 (2.0–4.3)	<0.001
Abu Raya [26]	PCS	Tdap -- (≥ 20)	no vac.	61/20	16.9 (10.5–27.0)	0.7 (0.3–1.8)	22.8 (8.8–58.7)	<0.001	61/20	17.81 (10.67–29.74)	1.12 (0.41–3.02)	15.9 (5.6–45.1)	<0.001
* (subset)*		*(27*–*36)*		*51/20*	*16.4 (9.6–28.0)*	*0.7 (0.3–1.8)*	*22.1 (8.1–60.0)*	*<0.001*	*51/20*	*17.3 (9.5–31.5)*	*1.12 (0.4–3.0)*	*15.4 (5.0–47.6)*	*<0.001*
* (subset)*		*(≥ 37)*		*7/20*	*28.1 (12.5–63.4)*	*0.7 (0.3–1.8)*	*38.0 (8.2–174.9)*	*<0.001*	*7/20*	*21.12 (7.9–56.2)*	*1.12 (0.4–3.0)*	*18.9 (3.3–108.1)*	*<0.001*
Hardy-Fairbanks [33]	PCS	Tdap – (anytime)	no vac.	5/53	14.3 (−-)	7.5 (−-)	1.9 (−-)	–	5/53	33.5 (−-)	12.6 (−-)	2.7 (−-)	–
Maertens [29]	PCS	Tdap 29 (22–33)	no vac.	56/41	31.4 (26.0–38.0)	6.4 (4.3–9.6)	4.9 (3.3–7.3)	<0.001	58/41	100.7 (82.0–123.0)	12.4 (8.0–19.0)	8.1 (5.3–12.5)	<0.001
Healy [34]	RCS	Tdap 9 (1–29)	before	19/86	10.5 (6.4–17.1)	14.0 (11.1–17.7)	0.8 (0.4–1.3)	0.29	19/86	17.3 (11.1–26.8)	16.7 (13.2–21.0)	1.0 (0.6–1.8)	0.90
Gall [5]	RCS	Tdap -- (anytime)	no vac.						52/52	Mean 28.2 (SE 2.8)	Mean 11.01 (SE 1.8)	MD 17.2 (10.7–23.8)	
FHA (EU or IU/ml)													
Munoz [31]	RCT	Tdap -- (30–32)	placebo	33/14	184.8 (142.8–239.1)	21.9 (10.9–44.1)	8.4 (4.8–15.0)	<0.001	31/14	234.2 (184.6–297.3)	25.1 (10.5–60.3)	9.3 (4.9–17.6)	<0.001
Hoang [30]	RCT	Tdap 26 (19–35)	TT	49/47	139.0 (109.0–176.0)	17.3 (14.0–21.4)	8.0 (5.8–11.0)	<0.001	49/46	93.0 (65.0–133.0)	27.3 (20.9–36.7)	3.4 (2.2–5.4)	<0.001
Abu Raya [26]	PCS	Tdap -- (≥ 20)	no vac.	61/20	187.4 (162.9–215.7)	13.4 (8.9–20.3)	14.0 (10.0–19.4)	<0.001	61/20	190.2 (160.9–224.8)	17.1 (10.2–28.7)	11.1 (7.4–16.6)	<0.001
* (subset)*		*(27*–*36)*		*51/20*	*192.0 (165.9–222.3)*	*13.4 (8.9–20.3)*	*14.3 (10.2–20.0)*	*<0.001*	*51/20*	*196.7 (163.4–236.9)*	*17.1 (10.2–28.7)*	*11.5 (7.5–17.6)*	*<0.001*
* (subset)*		*(≥ 37)*		*7/20*	*155.8 (109.3–222.2)*	*13.4 (8.9–20.3)*	*11.6 (5.7–23.7)*	*<0.001*	*7/20*	*138.0 (97.6–195.2)*	*17.1 (10.2–28.7)*	*8.1 (3.3–19.5)*	*<0.001*
Hardy-Fairbanks [33]	PCS	Tdap – (anytime)	no vac.	5/53	32.5 (−-)	9.6 (−-)	3.4 (−-)	–	5/53	66.1 (−-)	15.9 (−-)	4.2 (−-)	–
Maertens [29]	PCS	Tdap 29 (22–33)	no vac.	56/41	107.0 (91.0–126.0)	21.4 (16.6–27.5)	5.0 (3.8–6.6)	<0.001	58/41	140.0 (109.0–180.0)	27.5 (21.5–35.0)	5.1 (3.6–7.3)	<0.001
Healy [34]	RCS	Tdap 9 (1–29)	before	19/86	49.3 (28.4–85.8)	50.9 (40.6–63.9)	1.0 (0.6–1.7)	0.91	19/86	87.6 (56.3–136.4)	73.0 (57.6–92.6)	1.2 (0.7–2.1)	0.51
Gall [5]	RCS	Tdap -- (anytime)	no vac.						52/52	Mean 104.2 (SE 21.7)	Mean 26.8 (SE 4.0)	MD 77.3 (33.6–121.0)	<0.001
PRN (EU or IU/ml)													
Munoz [31]	RCT	Tdap -- (30–32)	placebo	45/35	184.5 (110.2–308.8)	12.2 (5.2–28.4)	15.1 (5.9–38.6)	<0.001	35/35	219.0 (134.4–357.0)	14.4 (5.4–38.4)	15.2 (5.9–39.3)	<0.001
Hoang [30]	RCT	Tdap 26 (19–35)	TT	49/48	111.0 (76.0–163.0)	9.4 (6.9–12.5)	11.8 (7.3–19.1)	<0.001	49/47	124 (86–179)	13.9 (10.5–18.2)	8.9 (5.6–14.1)	<0.001
Abu Raya [26]	PCS	Tdap -- (≥ 20)	no vac.	61/20	166.0 (125.7–219.4)	8.5 (3.5–20.3)	19.6 (10.0–38.6)	<0.001	61/20	162.1 (120.4–218.2)	10.6 (4.5–25.3)	15.3 (7.6–30.7)	<0.001
* (subset)*		*(27*–*36)*		*51/20*	*164.0 (119.5–225.1)*	*8.5 (3.5–20.3)*	*19.4 (9.4–39.9)*	*<0.001*	*51/20*	*161.5 (114.7–227.5)*	*10.6 (4.5–25.3)*	*15.2 (7.2–32.1)*	*<0.001*
* (subset)*		*(≥ 37)*		*7/20*	*181.6 (65.5–503.0)*	*8.5 (3.5–20.3)*	*21.5 (4.5–101.4)*	*<0.001*	*7/20*	*172.9 (68.7–434.8)*	*10.6 (4.5–25.3)*	*16.3 (3.5–75.0)*	*<0.001*
Hardy-Fairbanks [33]	PCS	Tdap -- (anytime)	no vac.	5/53	24.4 (−-)	6.4 (−-)	3.8 (−-)	–	5/53	48.5 (−-)	8.9 (−-)	5.5 (−-)	–
Maertens [29]	PCS	Tdap 29 (22–33)	no vac.	57/41	602.0 (485.5–747.0)	18.0 (13.0–24.0)	33.4 (23.4–47.9)	<0.001	57/41	697.0 (573.0–848.0)	21.0 (15.5–28.0)	33.2 (23.7–46.5)	<0.001
Healy [34]	RCS	Tdap 9 (1–29)	before	19/86	40.4 (18.9–87.3)	39.5 (28.3–55.0)	1.0 (0.5–2.2)	0.96	19/86	70 (32.5–150.5)	41.7 (81.6–4.07)	1.2 (0.5–2.6)	0.65
Gall [5]	RCS	Tdap -- (anytime)	no vac.						52/52	Mean 333.0 (SE 56.4)	Mean 24.7 (SE 5.8)	MD 308.3(195.8–420.0)	<0.001
FIM (EU or IU/ml)													
Munoz [31]	RCT	Tdap -- (30–32)	no vac.	11/38	1485.7 (979.9–2252.6)	34.9 (16.3–74.8)	42.6 (19.5–93.1)	<0.001	15/32	1867.0 (1211.7–2876.8)	51.8 (22.8–118)	36.0 (15.8–82.1)	<0.001
Hardy-Fairbanks [33]	PCS	Tdap -- (anytime)	no vac.	5/53	360.3 (−-)	17.7 (−-)	20.4 (−-)	–	5/53	912.9 (−-)	25.7 (−-)	35.5 (−-)	–
Healy [34]	RCS	Tdap 9 (1–29)	before	11/38	103.1 (42.7–249.0)	138.2 (97.2–196.5)	0.7 (0.3–1.7)	0.49	15/32	191.8 (84.5–435.7)	182.6 (127.7–261.2)	1.1 (0.5–2.4)	0.91
Gall [5]	RCS	Tdap -- (anytime)	no vac.	51/26					49/21	Mean 11,989.0 (SE 189.9)	Mean 82.8 (SE 14.59)	MD 1116.2 (738.3–1494.0)	<0.001

*RCT* randomised control trial, *PCS* prospective cohort study, *RCS* retrospective cohort study, *Tdap* a combined tetanus toxoid, reduced diphtheria toxoid, acellular pertussis vaccine, *TdaP-IPV* a combined tetanus, diphtheria, 5-component acellular pertussis, inactivated polio vaccine, *TT* tetanus toxoid (tetanus only vaccine), *SD* standard deviation, *MD* mean difference, *CI* confidence interval, *PT* pertussis toxin, *FHA* filamentous hemagglutinin, *PRN* pertactin, *FIM* fimbriae types 2 and 3; no vac, not receiving Tdap or pertussis-containing vaccine during pregnancy; before, Tdap before pregnancy; before, Tdap before pregnancy; −-, data not reported or not calculable. *1 EU = 1 IU

Table 3 shows the anti-pertussis antibody levels in infant blood prior to their first pertussis vaccinations at 2 months of age and after completion of the primary infant vaccination. At month two, one RCT [30] and two cohort studies [29, 33] showed significantly higher anti-pertussis antibody levels in infants of mothers vaccinated with Tdap during pregnancy compared with those of control infants.

**Table 3:** Geometric mean concentrations of pertussis antibodies in infants’ blood at ages two and 5 months

	Study design	Intervention/ exposure type, mean gestational week at vaccination (range)	Com-parator	Infant blood at 2 months of age (before primary vaccination)					Infant blood at 5 months of age (after primary vaccination				
				n: Vaccine /control	Vaccine Geometric mean (95% CI)	Control Geometric mean (95% CI)	Ratio of geometric means (95% CI)	p	n: Vaccine /control	Vaccine Geometric mean (95% CI)	Control Geometric mean (95% CI)	Ratio of geometric means (95% CI)	p
PT (EU or IU/ml)*													
Hoang [30]	RCT	Tdap 26 (19–35)	TT	45/35	4.2 (2.9–5.9)	0.8 (0.5–1.3)	5.3 (3.0–9.3)	<0.001	35/35	70 (58–84)	67 (53–84)	1.0 (0.8–1.4)	0.76
Hardy-Fairbanks [33]	PCS	Tdap -- (anytime)	no vac.	11/38	15.4 (−-)	4.8 (−-)	3.2 (−-)		15/32	56.8 (−-)	75.2 (−-)	0.8 (−-)	–
Maertens [29]	PCS	Tdap 29 (22–33)	no vac.	51/26	15.5 (12.1–20)	1.1 (0.7–1.6)	14.1 (9.0–22.1)	<0.001	49/21	29 (25–35)	54 (42–69)	0.5 (0.4–0.7)	<0.001
Ladhani [35]	RCS	TdaP/IPV -- (−-)	no vac.						129/203	28.8 (25.7–32.4)	43.2 (39.4–47.2)	0.7 (0.6–0.8)	<0.001
FHA (EU or IU /ml)													
Hoang [30]	RCT	Tdap 26 (19–35)	TT	45/35	59 (48–73)	23.1 (19.7–27)	2.6 (1.9–3.4)	<0.001	35/35	77 (66–90)	66.6 (56–78)	1.2 (0.9–1.4)	0.20
Hardy-Fairbanks [33]	PCS	Tdap -- (anytime)	no vac.	11/38	41.6 (−-)	5.6 (−-)	7.4 (−-)	–	15/32	61.4 (−-)	83.6 (−-)	0.7 (−-)	–
Maertens [29]	PCS	Tdap 29 (22–33)	no vac.	51/26	121 (100–145)	23 (19–27)	5.3 (4.0–7.0)	<0.001	49/21	65 (56–75)	54 (41–70)	1.2 (0.9–1.6)	0.19
Ladhani [35]	RCS	TdaP/IPV -- (−-)	no vac.						131/199	25.5 (23.0–28.3)	41.1 (37.5–45.1)	0.4 (0.2–0.6)	<0.001
PRN (EU or IU /ml)													
Hoang [30]	RCT	Tdap 26 (19–35)	TT	45/35	46 (32–66)	7.8 (6.6–9.4)	5.9 (3.8–9.1)	<0.001	35/35	83 (65–104)	132.6 (104–168)	0.6 (0.5–0.9)	0.006
Hardy-Fairbanks [33]	PCS	Tdap -- (anytime)	no vac.	11/38	32.1 (−-)	3.9 (−-)	8.2 (−-)	–	15/32	34.1 (−-)	50.7 (−-)	0.7 (−-)	–
Maertens [29]	PCS	Tdap 29 (22–33)	no vac.	51/26	253 (183–351)	17 (14.5–21)	14.9 (9.3–23.8)	<0.001	49/21	68 (56–84)	87 (62–121)	1.2 (0.9–23.8)	0.19
FIM (EU or IU /ml)													
Hardy-Fairbanks [33]	PCS	Tdap -- (anytime)	no vac.	11/38	296.4 (−-)	13.0 (−-)	22.8 (−-)	–	15/32	15.0 (−-)	10.0 (−-)	1.5 (−-)	–
Ladhani [35]	RCS	TdaP/IPV -- (−-)	no vac.						130/197	113.9 (99.0–131.1)	224.9 (196.1–258.0)	0.5 (0.2–1,3)	0.16

*RCT* randomised control trial, *PCS* prospective cohort study, *RCS* retrospective cohort study, *Tdap* a combined tetanus toxoid, reduced diphtheria toxoid, acellular pertussis vaccine. *TdaP-IPV* a combined tetanus, diphtheria, 5-component acellular pertussis, inactivated polio vaccine, *TT* tetanus toxoid (tetanus only vaccine), *SD* standard deviation, *MD* mean difference, *CI* confidence interval, *PT* pertussis toxin, *FHA* filamentous hemagglutinin, *PRN* pertactin, *FIM* fimbriae types 2 and 3; no vac, not receiving Tdap or pertussis-containing vaccine during pregnancy; before, Tdap before pregnancy; −-, data not reported or not calculable.*1 EU = 1 IU

At month five (1 month after the primary infant vaccination was completed), results were inconsistent, however. One RCT [30] reported that geometric mean concentrations of antibodies to pertactin but not to pertussis toxin or filamentous hemagglutinin were significantly (*p* = 0.006) lower in the Tdap group than in the tetanus toxoid group. One cohort study [29] showed that the geometric mean concentrations of antibodies to pertussis toxin but not to filamentous hemagglutinin or pertactin were significantly (*p* < 0.001) lower in the Tdap group compared with the unvaccinated group (*p* < 0.001). There was also one observational study [35] that showed that geometric mean concentrations of antibodies to pertussis toxin, filamentous hemagglutinin, and fimbriae types 2 and 3 in infants of TdaP/IPV-vaccinated mothers (recruited in 2013–2014) were significantly lower at 5 months of age (post-vaccination) (*p* < 0.001) than they were in infants in a historical cohort (recruited in 2011–2012) whose mothers did not receive a pertussis-containing vaccine during pregnancy. Based on GRADE, the quality of evidence was rated as moderate for the antibodies at birth but low for the antibodies at two and 5 months of age due to inconsistent results based on few studies as well as issues related to follow-up rate [30] and comparability [35].

##### Antibodies in breast milk

Two cohort studies [27] evaluated antibodies in breast milk, using ELISA kits (Euroimmun). One of these studies [28] evaluated anti-pertussis toxin secretory immunoglobulin A (IgA) at one time point (8–9 weeks postpartum), and another [27] evaluated IgA to pertussis toxin, filamentous hemagglutinin and IgG to pertussis toxin and filamentous hemagglutinin and pertactin in the colostrum and at two, four and 8 weeks postpartum. In the colostrum, there were statistically significant differences between the Tdap-vaccinated women compared to unvaccinated women in the geometric mean concentrations of IgA to filamentous hemagglutinin (one cohort study; *n* = 32; 24.12 EU/mL [95% CI 14.12–41.2, *n* = 21] vs. 6.52 EU/mL [95% CI 2.19–19.41]; *p* = 0.01) and IgG to pertactin (one cohort study; n = 32; 2.46 EU/mL [95% CI 1.19–5.11, n = 21] vs. <0.6 EU/mL; *p* = 0.03). In the breast milk at 2 weeks postpartum, the geometric mean concentrations of IgA to filamentous hemagglutinin remains significantly higher in the Tdap-vaccinated women compared with women with no vaccination (one cohort study; *n* = 30; 3.64 EU/mL [95% CI 2.4–5.51] vs. 1.37 EU/mL [95% CI 0.59–3.19]; *p* = 0.01]). At 4 weeks postpartum, there were no statistically significant differences in any of the measured antibodies in breast milk [27]. At 8–9 weeks postpartum, the results were inconsistent; one Belgium study [28] showed significantly higher levels of anti-pertussis IgA in the Tdap-vaccinated women compared with women with no vaccination for at least 5 years before delivery (*n* = 28; 0.55 EU/mL [95% CI 0.31–0.98] vs. 0.19 EU/mL [95% CI 0.16–0.23]; *p* = 0.01]), whereas one study in Israel [27] showed no statistically significant differences between the groups. The quality of the evidence for anti-pertussis antibodies in breast milk was rated as low using the GRADE criteria because one or two, small-sample observational study contributed to the outcome.

### Safety

#### Vaccine-related adverse outcomes

Table 4 shows the results of two RCTs [30, 31] that compared the incidence of adverse reaction outcomes between the Tdap vaccinated and the control groups. One RCT [31] conducted in the US showed statistically significant differences in the proportion of women who reported injection site reactions within 7 days after injection: women who had received the Tdap vaccine were more likely to complain injection site reactions (in particular pain), than the saline placebo group (*n* = 48; 26 [78.8%] of 33 in the vaccine group vs. 3 [20.0%] of 15 in the placebo group; RR 3.9 [95% CI 1.41–11.01]). Other adverse outcomes reported in the RCT [31] were erythema, swelling, and systemic reactions (fever - oral temperature ≥ 38 °C, headache, malaise, or myalgia), none of which showed statistically significant differences between the two groups. Another RCT [30] conducted in Vietnam reported that some women who received the vaccine experienced solicited adverse events such as stiffness, swelling and itching at the injection site, as well as fever and fatigue after injection, but there was no significant difference in the incidence between the Tdap vaccine group and the controls, i.e. women with the TT vaccine [30]. According to the GRADE criteria, the quality of the evidence for the vaccine related adverse outcomes was rated as moderate with results obtained by two RCTs.

**Table 4:** Safety of maternal antenatal pertussis vaccine

	Study design	Intervention/exposure type, mean gestational week at vaccination (range)	Compara-tor	Vaccine group		Control group		Crude RR (95% CI)	Adjusted RR (95% CI)
				Event n. (%)	N	Event n. (%)	N		
ADVERSE EVENTS (AE)									
Any solicited adverse events									
Hoang [30]^a^	RCT	Tdap 26 (19–35)	TT	23 (44.2)	52	22 (43.1)	51	1.0 (0.7–1.6)	
Munoz [31]^b^	RCT	Tdap -- (30–32)	placebo	26 (78.8)	33	5 (33.3)	15	2.4 (1.1–4.9)	
* Any injection site reactions*				*26 (78.8)*	*33*	*3 (20.0)*	*15*	*3.9 (1.4–11.0)*	
* (pain)*				*25 (75.8)*	*33*	*2 (13.3)*	*15*	*5.7 (1.5–20.9)*	
* (erythema/redness)*				*3 (9.1)*	*33*	*1 (6.7)*	*15*	*1.4 (0.2–12.1)*	
* (induration/swelling)*				*3 (9.1)*	*33*	*0 (0)*	*15*	NA^c^	
* Any systemic symptoms*				*12 (36.4)*	*33*	*3 (20.0)*	*15*	*1.8 (0.6–5.5)*	
* (fever)*				*1 (3.0)*	*33*	*0 (0)*	*15*	NA^c^	
* (headache)*				*11 (33.3)*	*33*	*3 (20.0)*	*15*	*1.7 (0.5–5.1)*	
* (malaise)*				*4 (12.1)*	*33*	*2 (13.3)*	*15*	*0.9 (0.2–4.4)*	
* (myalgia)*				*5 (15.2)*	*33*	*0 (0)*	*15*	NA^c^	
OBSTETRIC COMPLICATIONS									
Hypertensive disorders									
Donegan [32]	RCS	Any pertussis vaccine--(anytime)	no vaccine	22 (0.4)	6185	54 (0.3)	18,523	1.2 (0.7–2.0)	
Kharbanda [24]	RCS	Tdap, before 20	no vaccine	497 (8.2)	6083	7736 (8.0)	97,265	1.0 (0.9–1.1)	1.1 (1.0–1.2)^e^
Maertens [29]^d^	PCS	Tdap 29 (22–33)	no vaccine	6 (10.5)	57	2 (4.9)	41	2.2 (0.5–10.2)	
Premature contraction									
Hoang [30]	RCT	Tdap 26 (19–35)	TT	2 (3.8)	52	1 (2.0)	51	2.0 (0.2–21.0)	
Maertens [29]	PCS	Tdap 29 (22–33)	no vaccine	4 (7.0)	57	0 (0)	41	NA^c^	
Donegan [32]	RCS	Any pertussis vaccine--(anytime)	no vaccine	5 (0.1)	6185	21 (0.1)	18,523	0.7 (0.3–1.9)	
Chorioamnionitis									
Kharbanda [24]	RCS	Tdap -- (anytime)	no vaccine	1596 (6.1)	26,229	5329 (5.5)	97,265	1.11 (1.05–1.17)	1.2 (1.13–1.3)^e^
* (subset)*		*Tdap, 27*–*36*		*637 (0.1)*	*11,351*	*5329 (0.1)*	*97,265*	*1.02 (0.95–1.11)*	*1.1 (1.0–1.2)* ^*e*^
Proteinuria									
Kharbanda [25]^f^	RCS	Tdap -- (anytime)	no vaccine	84 (0.2)	53,885	207 (0.2)	109,253	0.8 (0.6–1.1)	0.8 (0.6–1.1)^g^
Caesarean section									
Donegan [32]	RCS	Any pertussis vaccine--(anytime)	no vaccine	1238 (20.0)	6185	3748 (20.2)	18,523	1.0 (0.9–1.0)	
Postpartum haemorrhage									
Donegan [32]	RCS	Any pertussis vaccine--(anytime)	no vaccine	59 (1.0)	6185	181 (1.0)	18,523	1.0 (0.7–1.3)	
Venous thromboembolism									
Kharbanda [25]^f^	RCS	Tdap -- (anytime)	no vaccine	22 (0.04)	53,885	69 (0.1)	109,253	0.6 (0.4–1.0)	0.7 (0.4–1.1)^g^
Thrombocytopenia									
Kharbanda [25]^f^	RCS	Tdap, ≥ 20	no vaccine	249 (0.1)	44,063	579 (0.1)	86,057	0.8 (0.7–1.0)	0.9 (0.7–1.0)^g^
Gestational diabetes									
Kharbanda [25]^f^	RCS	Tdap, ≥ 20	no vaccine	1101 (2.5)	44,063	2263 (2.6)	86,057	1.0 (0.9–1.0)	1.0 (0.9–1.0)^g^
Cardiac events									
Kharbanda [25]^f^	RCS	Tdap, ≥ 20	no vaccine	90 (0.2)	44,063	198 (0.2)	86,057	0.9 (0.7–1.1)	0.9 (0.7–1.2)^g^
Oligohydramnios									
Maertens [29]	PCS	Tdap 29 (22–33)	no Tdap	1 (1.8)	57	0 (0)	41	NA^c^	
Placenta praevia									
Maertens [29]	PCS	Tdap 29 wks (22–33)	no vaccine	0 (0)	57	1 (2.44)	41	0 (NA)^c^	
Donegan [32]	RCS	Any pertussis vaccine--(anytime)	no vaccine	2 (< 0.1)	6185	15 (0.1)	18,523	0.4 (0.1–1.7)	
PERINATAL COMPLICATIONS									
Stillbirth									
Hoang [30]	RCT	Tdap 26 (19–35)	TT	0 (0)	52	1 (2.0)	51	0 (NA)^c^	
Donegan [32]	RCS	Any pertussis vaccine--(anytime)	no vaccine	12 (0.2)	6185	42 (0.2)	18,523	0.9 (0.5–1.6)	
Shakib [36]	RCS	Tdap -- (anytime)	no vaccine	0 (0)	138	5 (0.9)	552	0 (NA)^c^	
Neonatal death									
Donegan [32]	RCS	Any pertussis vaccine--(anytime)	no vaccine	2 (<0.1)	6185	6 (<0.1)	18,523	1.0 (0.2–4.9)	
Preterm births									
Munoz [31]	RCT	Tdap -- (30–32)	no vaccine	3 (9.1)	33	1 (6.7)	15	1.4 (0.2–12.1)	
Hoang [30]	RCT	Tdap 26 (19–35)	TT	0 (0)	52	1 (2.0)	51	0 (NA)^c^	
Kharbanda [24]	RCS	Tdap -- (anytime)	no vaccine	1527 (5.8)	26,229	7544 (7.8)	97,265	0.8 (0.7–0.8)^h^	1.0 (1.0–1.1)^e^
Shakib [36]	RCS	Tdap -- (anytime)	no vaccine	8 (6.0)	134	38 (7.5)	505	0.8 (0.4–1.7)	
SGA (<10th percentile)									
Kharbanda [24]	RCS	Tdap -- (anytime)	no vaccine	2214 (8.4)	26,229	8086 (8.3)	97,265	1.0 (1.0–1.0)	1.0 (1.0–1.1)^e^
Low birth weight (< 2500 g)									
Donegan [32]	RCS	Any pertussis vaccine--(anytime)	no vaccine	126 (2.0)	6185	311 (1.7)	18,523	1.2 (1.0–1.5)	
Birth weight (kg)									
Munoz [29]	RCT	Tdap -- (30–32)	placebo	Mean 3.2 (SD 0.5)	33	Mean 3.5 (SD 0.7)	15	MD −0.3 (−0.7–0.1)	
Donegan [32]	RCS	Any pertussis vaccine--(anytime)	no vaccine	Median 3.5	6185	Median 3.5	18,523	Median difference 0	
Apgar scores 1 min									
Munoz [31]	RCT	Tdap -- (30–32)	no vaccine	Mean 8.0 (SD 1.4)	33	Mean 7.9 (SD 1.1)	15	MD 0.1(−0.07–0.9)	
Apgar scores 5 min									
Munoz [31]	RCT	Tdap -- (30–32)	no vaccine	Mean 8.9 (SD 0.2)	33	Mean 8.9 (SD 0.4)	15	MD 0 (−0.2–0.2)	
Abnormal conditions									
Munoz [31]	RCT	Tdap -- (30–32)	no vaccine	3 (9.1)^i^	33	3 (20.0)^j^	15	0.5 (0.1–2.0)	
Congenital anomalies									
Munoz [31]	RCT	Tdap -- (30–32)	no vaccine	1 (3.0)^k^	33	2 (13.3)^l^	15	0.2 (0.0–2.3)	

*RCT* Randomised control trial, *RCS* Retrospective cohort study, *RR* Risk ratio, *CI* Confidence interval, *MD* mean difference (associated CI calculated based on a t-distribution), *SD* standard deviation, *SGA* Small-for-gestational-age, *Tdap* tetanus toxoid, reduced diphtheria toxoid, acellular pertussis vaccine, *TT* tetanus toxoid; no vaccine, not receiving Tdap or pertussis-containing vaccine during pregnancy, *NA* not applicable

^a^Events occurred within 30 days after injection

^b^Events occurred within 7 days after injection

^c^Confidence interval cannot be calculated when one of the rates is zero

^d^Preterm and term pre-eclampsia, and hypertension counted as hypertensive disorders

^e^Adjusted for receipt of influenza vaccine, study site, and propensity score (which included sociodemographic characteristics [neighbourhood poverty index, age], presence of maternal comorbidities [hypertension, diabetes and cardiovascular or renal disease occurring prior to the start of pregnancy], receipt of medical care in the first trimester, and number of hospitalisations during the first 20 weeks of pregnancy as a surrogate of pregnancy complications)

^f^Events occurred within 42 days after injection

^g^Adjusted for patterns of care prior to vaccination date/index date, perinatal care utilisation (as measured with the Kotelchuck Adequacy of Prenatal Care Utilization Index) and having an inpatient encounter before the vaccination date/index date

^h^The crude risk ratio presented here is not the value the original authors reported. Instead, the researcher of this review recalculated it. This correction was made based on the numbers of events (preterm births) in both Tdap-vaccinated and unvaccinated groups, as reported in the original article

^i^Abnormal conditions at infant initial examination: Cephalohematoma (*n* = 2) and hydrocele (*n* = 1)

^j^Decreased breath sounds with increased anteroposterior diameter (*n* = 1), laceration (*n* = 1), large for gestational age and heart murmur (*n* = 1)

^k^Bilateral renal pelviectasis (*n* = 1)

1Asymptomatic atrial or ventricular septal defect (*n* = 1) and cardiomyopathy (*n* = 1)

#### Obstetric or perinatal complications

**Fig. 2:**
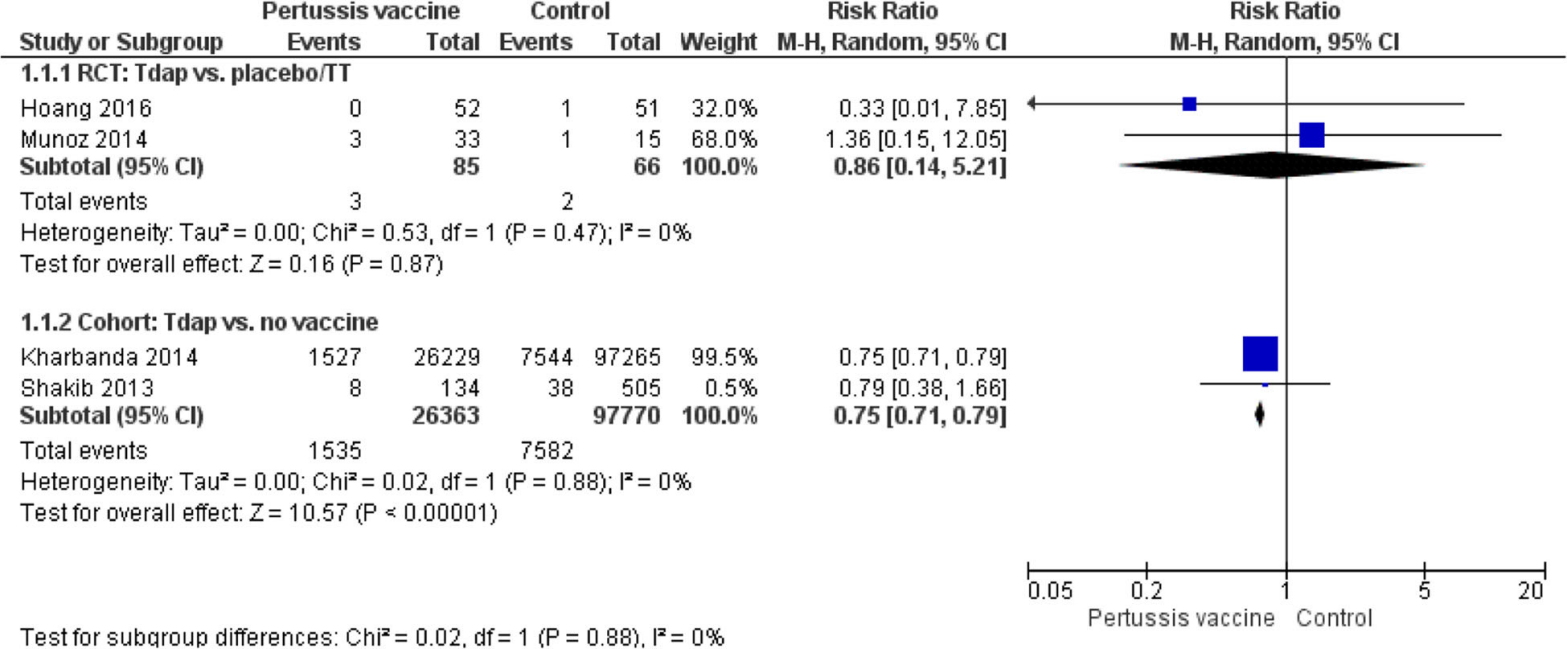
Incidence of preterm birth. Forest plots of studies with data on preterm birth as outcome. The first analysis included data from two RCTs with a total of 85 women in antental Tdap group and 66 women in the control. The second analysis included data from two cohort studies with a total of 26,363 women in antental Tdap group and 97,770 women in control with no pertussis vaccine. I-squared statistics for heterogeneity was 0% for both analyses

**Fig. 3:**
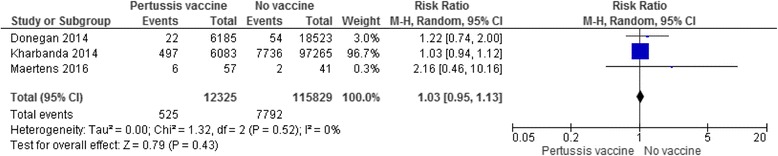
Incidence or prevalence of hypertensive disorder. Forest plot of studies with data on hypertensive disorder in mothers as outcome. The analysis included data from three cohort studies with a total of 12,325 mothers received a pertussis vaccine during pregnancy and 115,829 mothers who did not. I-squared statistics for heterogeneity was 0%

**Fig. 4:**
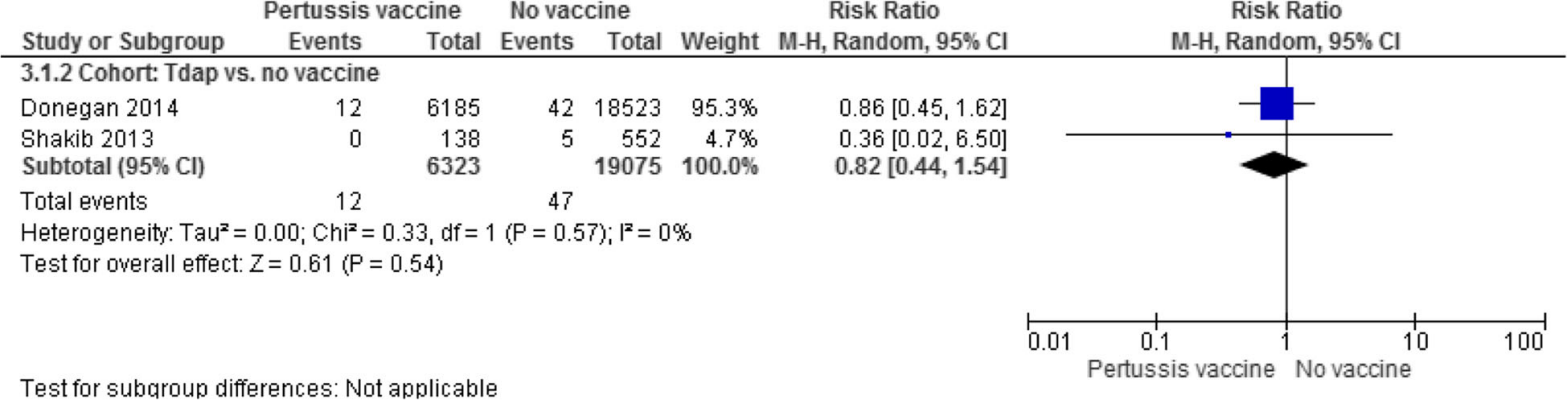
Incidence of stillbirth. Forest plot of studies with data on stillbirth as outcome. The analysis included data from two cohort studies with a total of 6323 babies whose mothers received a pertussis vaccine during pregnancy and 19,075 babies whose mothers did not receive the vaccine during pregnancy. I-squared statistics for heterogeneity was 0%

Table 4 presents a wide range of obstetric and perinatal complications that were evaluated in the included studies such as hypertensive disorder, chorioamnionitis, preterm birth, stillbirth and neonatal death. Results indicated that there was no increased risk for incidence of any of these complications in women who received the antenatal pertussis vaccine compared with women who did not. One exception was chorioamnionitis, for which one observational study [24] showed a higher risk in women who received the vaccine than in women who did not, even after adjustments by propensity scores were made for their baseline characteristics (e.g. socio-demographics and the presence of maternal comorbidities) (*n* = 123,494; 1596 [6.1%] of 26,229 in the Tdap-vaccinated group vs. 5326 [5.5%] of 972,653 in the unvaccinated group; adjusted RR 1.19 [95% CI: 1.13–1.26]). However, importantly, the study did not demonstrate the association between the antenatal pertussis vaccine and increased risk of preterm birth, a clinically important consequence of chorioamnionitis. Two additional RCTs [30, 31] and one observational study [36], in addition to [24], also reported no differences in incidence of preterm birth between the Tdap-vaccinated and unvaccinated groups (two RCTs: *n* = 151; 3 [3.5%] of 85 in the Tdap-vaccinated group vs. 2 [3.0%] of 66 in the unvaccinated group; RR 0.86 [95% CI 0.14–5.21]; two cohort studies: *n* = 124,133; 1535 [5.8%] of 26,365 in the vaccine group vs. 7582 [7.8%] of 97,770 in the control group; RR 0.75 [95% CI: 0.75–0.79], Fig. 2). In accordance with our prespecified outcomes, we also conducted meta-analyses where possible and presented forest plots which consistently show no significant difference in the incidence of hypertensive disorders in mothers (three cohort studies, *n* = 128,154; RR 1.03 [95% CI 0.95–1.13], Fig. 3) and stillbirth (i.e. intrauterine death after 24 weeks’ gestation; one RCT, *n* = 103, RR 0, meaning no stillbirth in the vaccine group; two cohort studies, *n* = 25,398; RR 0.82 [95% CI 0.44–1.54], Fig. 4). Only one study [32] evaluated neonatal death and small-for-gestational-age; the results were not statistically different in either outcome.

Following the GRADE criteria, the quality of the evidence for our prespecified obstetric or perinatal complications was rated as low for neonatal death and small-for-gestational-age because only one observational study contributed to the data; but moderate for hypertensive disorders, preterm birth and stillbirth because three different studies (including one RCT for preterm birth and stillbirth) showed consistent results.

## Discussion

### Summary of main findings

This review demonstrates that antenatal pertussis vaccination in middle to late pregnancy is associated with significantly higher antibody titres in maternal and infant blood than placebo or no vaccination. Studies testing maternal vaccinations at an early stage of pregnancy are small in number and hence evidence, if any for or against vaccination at an early stage of pregnancy, is scarce. Regarding safety concerns, there may be relatively minor injection site reactions, such as pain associated with the vaccine injection. However, the existing evidence has not demonstrated that having a maternal pertussis vaccination during pregnancy is associated with an increased risk of any serious maternal or perinatal complications such as hypertensive disorder, stillbirth, neonatal death or pre-term birth.

### Comparison with existing policies and guidelines

The UK Department of Health [39, 40] and the Centers for disease control and prevention (CDC) Advisory Committee on Immunization Practices (ACIP) [7] have both recommended administering the pertussis vaccine to pregnant women but the optimal timing suggested are different (at 16–38 weeks gestation according to the UK Department of Health and at 27–36 weeks gestation according to ACIP) to maximise maternal antibody response, passing more pertussis antibodies from mother to baby through intrauterine transfer, which in turn protects the infant from pertussis from the moment of birth [7]. Our review found that vaccination between 19 and 37 weeks of gestation was associated with higher antibody levels in maternal and foetal blood. However, there is a lack of evidence to suggest whether administering the vaccination during this time frame also reduces pertussis and severe pertussis-related complications in neonates because the relationship between antibody titres and prevention of pertussis-related complications is still not fully understood [41, 42]. Although, little is known regarding whether or to what extent the increased antibody concentrations transferred through the placenta have a protection effect for pertussis in neonates, vaccine effectiveness based on UK observational data is estimated to be around 90% [16, 43], possibly as a result of both passive antibodies and reduced maternal exposure to the infection. The results of the current review showing no evidence of increased risk of serious adverse outcomes reinforce the CDC’s recommendation of the pertussis vaccine during pregnancy based on its safety.

### Strength and limitations of the review

This is the first systematic review, to the authors’ knowledge, evaluating the efficacy and safety of pertussis vaccination during pregnancy. We developed a protocol (Additional file 4) prior to the review to avoid reporting bias. The literature search was comprehensive using multiple electronic databases and manual searches. This review included observational studies other than RCTs only, because it was anticipated that there would be few relevant studies — the antenatal pertussis vaccine programme was only introduced in 2011 in the US and in 2012 in the U.K. For the primary outcome of this review (pertussis infection in infants), there was no robust evidence from RCTs; the only evidence available was from one UK case-control study that examined the association between pertussis infection among infants and their mothers’ vaccination status during pregnancy, which limited the generalisability of the vaccine’s efficacy estimate to protect infants from pertussis in the wider population as incidences of pertussis vary across different populations and health care systems. The review included only studies written in English, but no study was excluded due to the reason of non-English publication. Some issues related to data (incorrect reporting, missing or insufficient data) might be further clarified if we had sought further data from the original researchers, which was not feasible given our limited resources.

#### Differences between protocol and review

There were situations in which we could not apply methods as planned in our protocol. Meta-analysis was not possible for nearly all outcomes because only a very small number of studies, sometimes only a single study, contributed to each outcome. Even in cases when two or more studies evaluated the same outcomes, it would not have been sensible to produce pooled estimates because the included studies differed in study design (RCT versus observational studies) or comparators (placebo versus unvaccinated during pregnancy versus vaccination before pregnancy). Some of the included studies reported an insufficient amount of background information. The lack of data also prohibited our plan to assess the interaction effects of the types and timings of vaccination and study settings through subgroup analyses. We were also not able to use sensitivity analysis to assess either study quality or potential bias caused by missing data. Finally, in this current review, we were not able to assess publication bias with a funnel plot because a small number of studies (n ranges from 1 to 3) contributed to each outcome. This was the case in which hypothesis testing for publication bias was deemed inappropriate due to the low power to detect real asymmetry [21].

## Conclusion

Recently, there has been a marked increase in the incidence of pertussis in the world, and newborn babies are at greatest risk of catching this highly infectious disease. Although pertussis vaccination in pregnancy is now recommended in several countries worldwide, pertussis vaccine has not yet been universally provided to the pregnant women. Our review findings aid evidence-based decision making for clinicians and public health commissioners, as well as for the women themselves regarding pertussis vaccination during pregnancy. Given that the existing evidence has not demonstrated that having a pertussis-containing vaccine during pregnancy is associated with an increased risk of any serious maternal and perinatal complications, maternal vaccination in pregnancy should continue to be supported, especially during the outbreak period, while further research endeavours to fill knowledge gaps and strengthen evidence of the vaccination’s efficacy and safety. Further studies need to focus on establishing the vaccine’s efficacy and effectiveness in relation to the prevention of pertussis and pertussis-related complications in infants through large, sufficiently powered, placebo-controlled RCTs. RCTs are also required to allow subgroup analyses that investigate whether vaccine efficacy differs according to the timing of vaccine administration, types of pertussis vaccination, and/or different settings. In particular, there is currently little evidence from low-income countries, where the risk of pertussis is higher and cases are more likely to be fatal; thus, the implications for clinical practice based on the findings of this review may be different [44]. There are also issues related to heterogeneity in the definitions of terms used to assess the safety of vaccinations that makes it difficult to compare vaccine trial results across different settings. Efforts have been made to establish a globally shared understanding of outcomes and to harmonise data collection by the Global Alignment of Immunization Safety Assessment in Pregnancy (GAIA) project supported by the Brighton Collaboration Foundation [45–48]. In line with these guidelines, ongoing monitoring is vital for the continued safety evaluation of the vaccine during pregnancy.

## Additional files

Additional file 1: Search strategies. Systematic electronic search stragegies for the CENTRAL, Medline, Embase and OpenGrey. (DOCX 20 kb)

Additional file 2: Excluded studies after full-texts retrieved and reasons for exclusion. A list of 76 excluded full-text articles with reasons for exclusion (DOCX 26 kb)

Additional file 3: Risk of bias assessment. The risk of bias assessment for all included studies using the Cochrane collaboration risk of bias tools (DOCX 16 kb)

Additional file 4: Efficacy and safety of pertussis vaccination for pregnant women – A systematic review of randomised controlled trials and observational studies (Protocol). A protocol developed prior to the review. (PDF 783 kb)

## Abbreviations

ACIP: Advisory committee on immunization practices
CCS: Case-control study
CENTRAL: Cochrane central register of controlled trials
CDC: Centers for disease control and prevention
CI: Confidence intervals
ELISA: Enzyme-linked immunosorbent assay
FHA: Filamentous hemagglutinin
FIM: Fimbriae types 2 and 3
GAIA: Global alignment of immunization safety assessment in pregnancy
GRADE: Grading of recommendations assessment, development and evaluation
IgA: Immunoglobulin A
IgG: Immunoglobulin G
IPV: Inactivated polio vaccine
MD: Mean difference
MeSH: Medical subject heading
OR: Odds ratio
PCS: Prospective cohort study
PRISMA: Preferred reporting items for systematic review and meta-analysis
PRN: Pertactin
PT: Pertussis toxin
RCS: Retrospective cohort study
RCT: Randomised controlled trial
RevMan: Review manager
RR: Risk ratio
SD: Standard deviation
Tdap: Tetanus-diphtheria-acellular pertussis
TT: Tetanus toxoid vaccine
