# Supplementary material for: Efficacy and safety of pertussis vaccination for pregnant women – a systematic review of randomised controlled trials and observational studies
Source: BMC Pregnancy Childbirth. 2017 Nov 22;17:390. doi: 10.1186/s12884-017-1559-2 (PMC5700667; doi:10.1186/s12884-017-1559-2)
Supplement: Supplementary file 1 — Search strategies. Systematic electronic search stragegies for the CENTRAL, Medline, Embase and OpenGrey. (DOCX 20 kb) [file 12884_2017_1559_MOESM1_ESM.docx]

**Additional File 1: Search strategies**

**Cochrane Central Register of Controlled Trials (CENTRAL)**

| #1 | neonat* |
| --- | --- |
| #2 | MeSH descriptor: [Postpartum Period] explode all trees |
| #3 | MeSH descriptor: [Infant, Newborn] explode all trees |
| #4 | MeSH descriptor: [Infant] this term only |
| #5 | maternal |
| #6 | matern* |
| #7 | perinatal |
| #8 | Pregnan* |
| #9 | MeSH descriptor: [Mothers] this term only |
| #10 | (#1 or #2 or #3 or #4 or #5 or #6 or #7 or #8 or #9) |
| #11 | MeSH descriptor: [Pertussis Vaccine] explode all trees |
| #12 | MeSH descriptor: [Vaccination] explode all trees |
| #13 | MeSH descriptor: [Immunization] this term only |
| #14 | immunisation |
| #15 | MeSH descriptor: [Immunization Programs] this term only |
| #16 | MeSH descriptor: [Diphtheria-Tetanus-Pertussis Vaccine] this term only |
| #17 | MeSH descriptor: [Diphtheria-Tetanus-acellular Pertussis Vaccines] this term only |
| #18 | Whole-cell pertussis vaccine |
| #19 | Tdap |
| #20 | dTap-IPV |
| #21 | (#11 or #12 or #13 or #14 or #15 or #16 or #17 or #18 or #19 or #20) |
| #22 | pertussis |
| #23 | MeSH descriptor: [Whooping Cough] this term only |
| #24 | MeSH descriptor: [Antibodies, Bacterial] explode all trees |
| #25 | anti-pertussis antibodies |
| #26 | IgG |
| #27 | MeSH descriptor: [Immunoglobulin G] explode all trees |
| #28 | IgA |
| #29 | MeSH descriptor: [Immunoglobulin A] explode all trees |
| #30 | MeSH descriptor: [Antigen-Antibody Reactions] explode all trees |
| #31 | MeSH descriptor: [Drug-Related Side Effects and Adverse Reactions] explode all trees |
| #32 | MeSH descriptor: [Pre-Eclampsia] explode all trees |
| #33 | MeSH descriptor: [Eclampsia] explode all trees |
| #34 | MeSH descriptor: [Hypertension, Pregnancy-Induced] explode all trees |
| #35 | MeSH descriptor: [Pregnancy Outcome] explode all trees |
| #36 | MeSH descriptor: [Pregnancy Complications] explode all trees |
| #37 | MeSH descriptor: [Puerperal Disorders] explode all trees |
| #38 | MeSH descriptor: [Infant, Premature] explode all trees |
| #39 | MeSH descriptor: [Infant, Small for Gestational Age] explode all trees |
| #40 | (#22 or #23 or #24 or #25 or #26 or #27 or #28 or #29 or #30 or #31 or #32 or #33 or #34 or #35 or #36 or #37 or #38 or #39) |
| #41 | (#10 and #21 and #40) |

**(Additional File 1 cont.)**

**Medline (OvidSp)**

| 1 | maternal.mp. [mp=title, abstract, original title, name of substance word, subject heading word, keyword heading word, protocol supplementary concept word, rare disease supplementary concept word, unique identifier] |
| --- | --- |
| 2 | matern*.mp. |
| 3 | perinatal.mp. |
| 4 | Pregnan*.mp. |
| 5 | mother.mp. |
| 6 | exp Mothers/ |
| 7 | 1 or 2 or 3 or 4 or 5 or 6 |
| 8 | exp Pertussis Vaccine/ |
| 9 | vaccination*.mp. |
| 10 | exp Vaccination/ |
| 11 | exp Vaccines/ |
| 12 | exp Vaccines, Inactivated/ |
| 13 | exp Vaccines, Acellular/ |
| 14 | Immuni?ation*.mp. |
| 15 | immunisation.mp. |
| 16 | Immunization/ |
| 17 | exp Immunization Programs/ |
| 18 | exp Diphtheria-Tetanus-Pertussis Vaccine/ |
| 19 | exp Diphtheria-Tetanus-acellular Pertussis Vaccines/ |
| 20 | Whole-cell pertussis vaccine.mp. |
| 21 | Tdap.mp. |
| 22 | dTap-IPV.mp. |
| 23 | 8 or 9 or 10 or 11 or 12 or 13 or 14 or 15 or 16 or 17 or 18 or 19 or 20 or 21 or 22 |
| 24 | pertussis.mp. or exp Whooping Cough/ |
| 25 | exp Antibodies, Bacterial/ |
| 26 | anti-pertussis antibodies.mp. |
| 27 | IgG.mp. |
| 28 | exp Immunoglobulin G/ |
| 29 | IgA.mp. |
| 30 | exp Immunoglobulin A/ |
| 31 | exp Antigen-Antibody Reactions/ |
| 32 | exp "Drug-Related Side Effects and Adverse Reactions"/ |
| 33 | exp Pre-Eclampsia/ |
| 34 | exp Eclampsia/ |
| 35 | exp Hypertension, Pregnancy-Induced/ |
| 36 | exp Pregnancy Outcome/ |
| 37 | exp Pregnancy Complications/ |
| 38 | exp Puerperal Disorders/ |
| 39 | exp Infant, Premature/ |
| 40 | exp Infant, Small for Gestational Age/ |
| 41 | 24 or 25 or 26 or 27 or 28 or 29 or 30 or 31 or 32 or 33 or 34 or 35 or 36 or 37 or 38 or 39 or 40 |
| 42 | 7 and 23 and 41 |

**(Additional File 1 cont.)**

**Embase**

| 1 | maternal.mp. [mp=title, abstract, subject headings, heading word, drug trade name, original title, device manufacturer, drug manufacturer, device trade name, keyword] |
| --- | --- |
| 2 | matern*.mp. |
| 3 | perinatal.mp. |
| 4 | exp pregnancy/ or exp pregnant woman/ or pregnant.mp. |
| 5 | exp expectant mother/ or exp mother/ |
| 6 | 1 or 2 or 3 or 4 or 5 |
| 7 | exp diphtheria pertussis tetanus Haemophilus influenzae type b vaccine/ or exp pertussis vaccine/ or exp diphtheria pertussis poliomyelitis tetanus hepatitis B vaccine/ or exp diphtheria pertussis tetanus hepatitis B vaccine/ or exp diphtheria pertussis poliomyelitis tetanus Haemophilus influenzae type b vaccine/ or exp diphtheria pertussis tetanus vaccine/ or exp diphtheria pertussis poliomyelitis tetanus vaccine/ or exp diphtheria pertussis poliomyelitis tetanus Haemophilus influenzae type b hepatitis B vaccine/ or exp pertussis toxin/ or exp diphtheria pertussis tetanus Haemophilus influenzae type b hepatitis B vaccine/ |
| 8 | exp acellular vaccine/ or exp bacterial vaccine/ or vaccine/ |
| 9 | exp inactivated vaccine/ |
| 10 | exp vaccination/ |
| 11 | immunisation.mp. or exp immunization/ |
| 12 | Tdap.mp. |
| 13 | dTap-IPV.mp. |
| 14 | 7 or 8 or 9 or 10 or 11 or 12 or 13 |
| 15 | whooping cough.mp. or exp pertussis/ |
| 16 | exp antibody response/ or exp immune response/ |
| 17 | exp bacterium antibody/ |
| 18 | exp immunoglobulin/ or exp immunoglobulin G/ or exp "antibody and immunoglobulin structure, function and production"/ or exp immunoglobulin A antibody/ or exp "antibody and immunoglobulin production"/ or exp immunoglobulin G antibody/ or exp immunoglobulin G deficiency/ or exp immunoglobulin A/ |
| 19 | IgA.mp. |
| 20 | IgG.mp. |
| 21 | exp adverse drug reaction/ or exp side effect/ or exp drug effect/ |
| 22 | exp "eclampsia and preeclampsia"/ or exp eclampsia/ |
| 23 | exp preeclampsia/ |
| 24 | exp hypertension/ |
| 25 | exp low birth weight/ or exp premature labor/ |
| 26 | exp small for date infant/ |
| 27 | exp prematurity/ |
| 28 | 15 or 16 or 17 or 18 or 19 or 20 or 21 or 22 or 23 or 24 or 25 or 26 or 27 |
| 29 | 6 and 14 and 28 |

**(Additional File 1 cont.)**

**OpenGray**

- “Bordetella pertussis”
- “Bordetella pertussis” AND Vaccine
- “Bordetella pertussis” AND Vaccination
- “Whooping cough” AND Vaccine
- “Whooping cough” AND Vaccination
