# Supplementary material for: Efficacy and safety of pertussis vaccination for pregnant women – a systematic review of randomised controlled trials and observational studies
Source: BMC Pregnancy Childbirth. 2017 Nov 22;17:390. doi: 10.1186/s12884-017-1559-2 (PMC5700667; doi:10.1186/s12884-017-1559-2)
Supplement: Supplementary file 2 — Excluded studies after full-texts retrieved and reasons for exclusion. A list of 76 excluded full-text articles with reasons for exclusion (DOCX 26 kb) [file 12884_2017_1559_MOESM2_ESM.docx]

## Additional File 2: Excluded studies after full-texts retrieved and the reason for the exclusion

| **Pertussis or pertussis-containing vaccine not administered during pregnancy or information of maternal vaccination status during pregnancy was not reported** |
| --- |
| BIGHAM, M., KONRAD, S., VAN BUYNDER, P., VAN BUYNDER, J., ISAAC-RENTON, J., ELSHERIF, M. & HALPERIN, S. A. 2014. Low pertussis toxin antibody levels in two regional cohorts of Canadian pregnant women. Vaccine, 32, 6493-6498.  BURSTYN, D. G., BARAFF, L. J. & PEPPLER, M. S. 1983. Serological response to filamentous hemagglutinin and lymphocytosis-promoting toxin of Bordetella pertussis. Infection and Immunity, 41, 1150-1156.  DE GREEFF, S. C., DE MELKER, H. E., WESTERHOF, A., SCHELLEKENS, J. F. P., MOOI, F. R. & VAN BOVEN, M. 2012. Estimation of household transmission rates of pertussis and the effect of cocooning vaccination strategies on infant pertussis. Epidemiology, 23, 852-860.  DE GREEFF, S. C., MOOI, F. R., WESTERHOF, A., VERBAKEL, J. M., PEETERS, M. F., HEUVELMAN, C. J., NOTERMANS, D. W., ELVERS, L. H., SCHELLEKENS, J. F. & DE MELKER, H. E. 2010. Pertussis disease burden in the household: how to protect young infants. Clinical Infectious Diseases, 50, 1339-45.  EDWARDS, K. M. 2003. Pertussis: an important target for maternal immunization. Vaccine, 21, 3483-6.  ERCAN, T. E., SONMEZ, C., VURAL, M., ERGINOZ, E., TORUNOGLU, M. A. & PERK, Y. 2013. Seroprevalance of pertussis antibodies in maternal and cord blood of preterm and term infants. Vaccine, 31, 4172-6.  ESSERY, S. D., RAZA, M. W., ZORGANI, A., MACKENZIE, D. A., JAMES, V. S., WEIR, D. M., BUSUTTIL, A., HALLAM, N. & BLACKWELL, C. 1999. The protective effect of immunisation against diphtheria, pertussis and tetanus (DPT) in relation to sudden infant death syndrome. FEMS Immunology & Medical Microbiology, 25, 183-92.  FALLO, A., MANONELLES, G., HOZBOR, D., LARA, C., HUESPE, M., MAZZEO, S., CANLE, O., GALAS, M. & LOPEZ, E. 2014. Pertussis seroprevalence in adults, post-partum women and umbilical cord blood. Archivos Argentinos de Pediatria, 12, 315-322.  GONIK, B., PUDER, K. S., GONIK, N. & KRUGER, M. 2005. Seroprevalence of Bordetella pertussis antibodies in mothers and their newborn infants. Infectious Diseases in Obstetrics & Gynecology, 13, 59-61.  HABERLING, D. L., HOLMAN, R. C., PADDOCK, C. D. & MURPHY, T. V. 2009. Infant and maternal risk factors for pertussis-related infant mortality in the United States, 1999 to 2004. Pediatric Infectious Disease Journal, 28, 194-8.  HASHEMI, S. H., ZAMANI, M., MAMANI, M., JAVEDANPOOR, R., RAHIGHI, A. H. & NADI, E. 2014. Seroprevalence of Bordetella pertussis antibody in pregnant women in Iran. Journal of Research in Health Sciences, 14, 128-131.  HEALY, C. M., MUNOZ, F. M., RENCH, M. A., HALASA, N. B., EDWARDS, K. M. & BAKER, C. J. 2004. Prevalence of pertussis antibodies in maternal delivery, cord, and infant serum. Journal of Infectious Diseases, 190, 335-40.  HEININGER, U., RIFFELMANN, M., BAR, G., RUDIN, C. & VON KONIG, C. H. W. 2013. The protective role of maternally derived antibodies against bordetella pertussis in young infants. Pediatric Infectious Disease Journal, 32, 695-698.  HEININGER, U., RIFFELMANN, M., LEINEWEBER, B. & WIRSING VON KOENIG, C. H. 2009. Maternally derived antibodies against Bordetella pertussis antigens pertussis toxin and filamentous hemagglutinin in preterm and full term newborns. Pediatric Infectious Disease Journal, 28, 443-5.  IZURIETA, H. S., KENYON, T. A., STREBEL, P. M., BAUGHMAN, A. L., SHULMAN, S. T. & WHARTON, M. 1996. Risk factors for pertussis in young infants during an outbreak in Chicago in 1993. Clinical Infectious Diseases, 22, 503-7.  JONES, C., POLLOCK, L., BARNETT, S. M., BATTERSBY, A. & KAMPMANN, B. 2013. Specific antibodies against vaccine-preventable infections: A mother-infant cohort study. BMJ Open, 3.  JONES, C., POLLOCK, L., BARNETT, S. M., BATTERSBY, A. & KAMPMANN, B. 2014. The relationship between concentration of specific antibody at birth and subsequent response to primary immunization. Vaccine, 32, 996-1002.  LEURIDAN, E., HENS, N., PEETERS, N., DE WITTE, L., VAN DER MEEREN, O. & VAN DAMME, P. 2011. Effect of a prepregnancy pertussis booster dose on maternal antibody titers in young infants. Pediatric Infectious Disease Journal, 30, 608-10.  MARSHALL, H., CLARKE, M., RASIAH, K., RICHMOND, P., BUTTERY, J., REYNOLDS, G., ANDREWS, R., NISSEN, M., WOOD, N. & MCINTYRE, P. 2015. Predictors of disease severity in children hospitalized for pertussis during an epidemic. Pediatric Infectious Disease Journal, 34, 339-45.  MIKELOVA, L. K., HALPERIN, S. A., SCHEIFELE, D., SMITH, B., FORD-JONES, E., VAUDRY, W., JADAVJI, T., LAW, B., MOORE, D. & MEMBERS OF THE IMMUNIZATION MONITORING PROGRAM, A. 2003. Predictors of death in infants hospitalized with pertussis: a case-control study of 16 pertussis deaths in Canada. Journal of Pediatrics, 143, 576-81.  QUINELLO, C., QUINTILIO, W., CARNEIRO-SAMPAIO, M. & PALMEIRA, P. 2010. Passive acquisition of protective antibodies reactive with bordetella pertussis in newborns via placental transfer and breast-feeding. Scandinavian Journal of Immunology, 72, 66-73.  ROTHSTEIN, E. P., ANDERSON, E. L., DECKER, M. D., POLAND, G. A., REISINGER, K. S., BLATTER, M. M., JACOBSON, R. M., MINK, C. A., GENNEVOIS, D., IZU, A. E., SINANGIL, F. & LANGENBERG, A. G. 1999. An acellular pertussis vaccine in healthy adults: safety and immunogenicity. Vaccine, 17, 2999-3006.  SMALLENBURG, L. C. S., VAN WELIE, N. A., ELVERS, L. H., VAN HUISSELING, J. C. M., TEUNIS, P. F. M. & VERSTEEGH, F. G. A. 2014. Decline of IgG pertussis toxin measured in umbilical cord blood, and neonatal and early infant serum. European Journal of Clinical Microbiology and Infectious Diseases, 33, 1541-1545.  TERRANELLA, A., ASAY, G. R. B., MESSONNIER, M. L., CLARK, T. A. & LIANG, J. L. 2013. Pregnancy dose Tdap and postpartum cocooning to prevent infant pertussis: A decision analysis. Pediatrics, 131, e1748-e1756.  VAN DEN BERG, J. P., WESTERBEEK, E. A. M., BERBERS, G. A. M., VAN GAGELDONK, P. G. M., VAN DER KLIS, F. R. M. & VAN ELBURG, R. M. 2010. Transplacental transport of IgG antibodies specific for pertussis, diphtheria, tetanus, haemophilus influenzae type b, and neisseria meningitidis serogroup C is lower in preterm compared with term infants. Pediatric Infectious Disease Journal, 29, 801-805.  VAN HOEK, A. J., CAMPBELL, H., AMIRTHALINGAM, G., ANDREWS, N. & MILLER, E. 2013. The number of deaths among infants under one year of age in England with pertussis: Results of a capture/recapture analysis for the period 2001 to 2011. Eurosurveillance, 18.  WINTER, K., HARRIMAN, K., ZIPPRICH, J., SCHECHTER, R., TALARICO, J., WATT, J. & CHAVEZ, G. 2012. California pertussis epidemic, 2010. Journal of Pediatrics, 161, 1091-6.  ZAMIR, C. S., DAHAN, D. B. & SHOOB, H. 2015. Pertussis in infants under one year old: risk markers and vaccination status--a case-control study. Vaccine, 33, 2073-8. |
| **Efficacy/safety of pertussis vaccine during pregnancy not examined** |
| AHMAD, S. M., ALAM, J., AFSAR, N. A., HUDA, N., KABIR, Y., QADRI, F., RAQIB, R. & STEPHENSEN, C. B. 2016. Comparisons of the effect of naturally acquired maternal pertussis antibodies and antenatal vaccination induced maternal tetanus antibodies on infant's antibody secreting lymphocyte responses and circulating plasma antibody levels. Human vaccines & Immunotherapeutics, 12, 886-93.  FRERE, J., DE WALS, P., OVETCHKINE, P., COIC, L., AUDIBERT, F. & TAPIERO, B. 2013. Evaluation of several approaches to immunize parents of neonates against B. pertussis. Vaccine, 31, 6087-91.  GOLDFARB, I. T., LITTLE, S., BROWN, J. & RILEY, L. E. 2014. Use of the combined tetanus-diphtheria and pertussis vaccine during pregnancy. American Journal of Obstetrics & Gynecology, 211, 299.e1-5.  HEALY, C. M., RENCH, M. A., WOOTTON, S. H. & CASTAGNINI, L. A. 2015. Evaluation of the impact of a pertussis cocooning program on infant pertussis infection. Pediatric Infectious Disease Journal, 34, 22-26.  HOUSEY, M., ZHANG, F., MILLER, C., LYON-CALLO, S., MCFADDEN, J., GARCIA, E., POTTER, R., CENTERS FOR DISEASE, C. & PREVENTION 2014. Vaccination with tetanus, diphtheria, and acellular pertussis vaccine of pregnant women enrolled in Medicaid--Michigan, 2011-2013. MMWR - Morbidity & Mortality Weekly Report, 63, 839-42.  KOEPKE, R., EICKHOFF, J. C., AYELE, R. A., PETIT, A. B., SCHAUER, S. L., HOPFENSPERGER, D. J., CONWAY, J. H. & DAVIS, J. P. 2014. Estimating the effectiveness of tetanus-diphtheria-acellular pertussis vaccine (Tdap) for preventing pertussis: evidence of rapidly waning immunity and difference in effectiveness by Tdap brand. J Infect Dis, 210, 942-53.  MARTIN, S. W., PAWLOSKI, L., WILLIAMS, M., WEENING, K., DEBOLT, C., QIN, X., REYNOLDS, L., KENYON, C., GIAMBRONE, G., KUDISH, K., MILLER, L., SELVAGE, D., LEE, A., SKOFF, T. H., KAMIYA, H., CASSIDAY, P. K., TONDELLA, M. L. & CLARK, T. A. 2015. Pertactin-negative Bordetella pertussis strains: evidence for a possible selective advantage. Clinical Infectious Diseases, 60, 223-7.  MORGAN, J. L., BAGGARI, S. R., CHUNG, W., RITCH, J., MCINTIRE, D. D. & SHEFFIELD, J. S. 2015. Association of a Best-Practice Alert and Prenatal Administration With Tetanus Toxoid, Reduced Diphtheria Toxoid, and Acellular Pertussis Vaccination Rates. Obstetrics & Gynecology, 126, 333-7.  ZAMIR, C. S., DAHAN, D. B. & SHOOB, H. 2015. Pertussis in infants under one year old: risk markers and vaccination status--a case-control study. Vaccine, 33, 2073-8. |
| **Outcomes of interest not examined or not obtainable (studies only gave pertussis positive but not pertussis negative or the denomination, so the incidence or prevalence could not be calculated)** |
| ABU RAYA, B., SRUGO, I., KESSEL, A., PETERMAN, M., VAKNIN, A. & BAMBERGER, E. 2015. The decline of pertussis-specific antibodies after tetanus, diphtheria, and acellular pertussis immunization in late pregnancy. Journal of Infectious Diseases, 212, 1869-1873.  AMIRTHALINGAM, G., ANDREWS, N., CAMPBELL, H., RIBEIRO, S., KARA, E., DONEGAN, K., FRY, N. K., MILLER, E. & RAMSAY, M. 2014. Effectiveness of maternal pertussis vaccination in England: An observational study. The Lancet, 384, 1521-1528.  LUGNER, A. K., VAN DER MAAS, N., VAN BOVEN, M., MOOI, F. R. & DE MELKER, H. E. 2013. Cost-effectiveness of targeted vaccination to protect new-borns against pertussis: comparing neonatal, maternal, and cocooning vaccination strategies. Vaccine, 31, 5392-7.  PETERS, T. R., BANKS, G. C., SNIVELY, B. M. & POEHLING, K. A. 2012. Potential impact of parental Tdap immunization on infant pertussis hospitalizations. Vaccine, 30, 5527-32.  ZHETEYEVA, Y. A., MORO, P. L., TEPPER, N. K., RASMUSSEN, S. A., BARASH, F. E., REVZINA, N. V., KISSIN, D., LEWIS, P. W., YUE, X., HABER, P., TOKARS, J. I., VELLOZZI, C. & BRODER, K. R. 2012. Adverse event reports after tetanus toxoid, reduced diphtheria toxoid, and acellular pertussis vaccines in pregnant women. American Journal of Obstetrics & Gynecology, 207, 59.e1-7. |
| **No control group** **of women without pertussis (pertussis containing) vaccine in pregnancy; or vaccine status in the control uncertain** |
| EBERHARDT, C. S., BLANCHARD-ROHNER, G., LEMAITRE, B., BOUKRID, M., COMBESCURE, C., OTHENIN-GIRARD, V., CHILIN, A., PETRE, J., DE TEJADA, B. M. & SIEGRIST, C. A. 2016. Maternal Immunization Earlier in Pregnancy Maximizes Antibody Transfer and Expected Infant Seropositivity Against Pertussis. Clinical Infectious Diseases, 62, 829-36.  HUYGEN, K., CABORE, R. N., MAERTENS, K., VAN DAMME, P. & LEURIDAN, E. 2015. Humoral and cell mediated immune responses to a pertussis containing vaccine in pregnant and nonpregnant women. Vaccine, 33, 4117-23.  MORO, P. L., CRAGAN, J., TEPPER, N., ZHETEYEVA, Y., MUSERU, O., LEWIS, P. & BRODER, K. 2016. Enhanced surveillance of tetanus toxoid, reduced diphtheria toxoid, and acellular pertussis (Tdap) vaccines in pregnancy in the Vaccine Adverse Event Reporting System (VAERS), 2011-2015. Vaccine, 34, 2349-53.  PETOUSIS-HARRIS, H., WALLS, T., WATSON, D., PAYNTER, J., GRAHAM, P. & TURNER, N. 2016. Safety of Tdap vaccine in pregnant women: an observational study. BMJ Open, 6, e010911.  SUKUMARAN, L., MCCARTHY, N. L., KHARBANDA, E. O., MCNEIL, M. M., NALEWAY, A. L., KLEIN, N. P., JACKSON, M. L., HAMBIDGE, S. J., LUGG, M. M., LI, R., WEINTRAUB, E. S., BEDNARCZYK, R. A., KING, J. P., DESTEFANO, F., ORENSTEIN, W. A. & OMER, S. B. 2015a. Association of tdap vaccination with acute events and adverse birth outcomes among pregnant women with prior tetanuscontaining immunizations. JAMA - Journal of the American Medical Association, 314, 1581-1587.  SUKUMARAN, L., MCCARTHY, N. L., KHARBANDA, E. O., WEINTRAUB, E. S., VAZQUEZ-BENITEZ, G., MCNEIL, M. M., LI, R., KLEIN, N. P., HAMBIDGE, S. J., NALEWAY, A. L., LUGG, M. M., JACKSON, M. L., KING, J. P., DESTEFANO, F., OMER, S. B. & ORENSTEIN, W. A. 2015b. Safety of Tetanus Toxoid, Reduced Diphtheria Toxoid, and Acellular Pertussis and Influenza Vaccinations in Pregnancy. Obstetrics & Gynecology, 126, 1069-74.  TALBOT, E. A., BROWN, K. H., KIRKLAND, K. B., BAUGHMAN, A. L., HALPERIN, S. A. & BRODER, K. R. 2010. The safety of immunizing with tetanus-diphtheria-acellular pertussis vaccine (Tdap) less than 2 years following previous tetanus vaccination: Experience during a mass vaccination campaign of healthcare personnel during a respiratory illness outbreak. Vaccine, 28, 8001-7.  VILAJELIU, A., GONCE, A., LOPEZ, M., COSTA, J., ROCAMORA, L., RIOS, J., TEIXIDO, I., BAYAS, J. M., AVILES, M., ARRANZ, A., BELLART, J., BOMBI, T., CASALS, E., CASTELLVI, L., COBO, M. T., FERRER, L., FIGUERAS, F., GARCETE, L. A., OTERO, L. G., GOMEZ, O., GONZALEZ, E. R., GONZALEZ, E., HERNANDEZ, A. S., LANNA, A., MARTINEZ, M., MERCADE, I., MIGLIORELLI, F., MULA, R., MUNOZ, M., MUNROS, J., PALACIO, M., PEGUERO, A., PERICOT, A., RAMIREZ, J. C., RIUS, M., SOLERNOU, R., SOVERAL, I. & TORRES, X. 2015. Combined tetanus-diphtheria and pertussis vaccine during pregnancy: Transfer of maternal pertussis antibodies to the newborn. Vaccine, 33, 1056-1062.  WALLS, T., GRAHAM, P., PETOUSIS-HARRIS, H., HILL, L. & AUSTIN, N. 2016. Infant outcomes after exposure to Tdap vaccine in pregnancy: an observational study. BMJ Open, 6, e009536. |
| **Review/overview /reports** |
| AMIRTHALINGAM, G. 2013. Strategies to control pertussis in infants. Archives of Disease in Childhood, 98, 552-5.  CENTERS FOR DISEASE. 2013. Updated recommendations for use of tetanus toxoid, reduced diphtheria toxoid, and acellular pertussis vaccine (Tdap) in pregnant women - Advisory committee on immunization practices (ACIP), 2012. Morbidity and Mortality Weekly Report, 62, 131-135.  FRENCH, S. & MACKILLOP, L. 2013. Vaccinations in pregnancy. Obstetrics, Gynaecology and Reproductive Medicine, 23, 38-44.  HOGAN, J., FRENCH, S. & MACKILLOP, L. 2016. Vaccinations in pregnancy. Obstetrics, Gynaecology and Reproductive Medicine, 26, 72-79.  HUNTER, K. 2013. Helping prevent the spread of pertussis. Nursing New Zealand (Wellington), 19, 26-8.  LEURIDAN, E., HENS, N., PEETERS, N., DE WITTE, L., VAN DER MEEREN, O. & VAN DAMME, P. 2011. Effect of a prepregnancy pertussis booster dose on maternal antibody titers in young infants. Pediatric Infectious Disease Journal, 30, 608-10.  MATLOW, J. N., PUPCO, A., BOZZO, P. & KOREN, G. 2013. Tdap vaccination during pregnancy to reduce pertussis infection in young infants. Canadian Family Physician, 59, 497-8.  NITSCH-OSUCH, A., KORZENIEWSKI, K., GAWLAK, M., ZYCINSKA, K., WARDYN, K. & KUCHAR, E. 2015. Epidemiological and clinical reasons for vaccination against pertussis and influenza in pregnant women. Advances in Experimental Medicine & Biology, 849, 11-21.  SWAMY, G. K. & BEIGI, R. H. 2015. Maternal benefits of immunization during pregnancy. Vaccine, 33, 6436-6440. |
| **Systematic review not examined efficacy of pertussis vaccine/systematic review protocol only** |
| CASSIDY, C., MACDONALD, N. E., STEENBEEK, A. & TOP, K. A. 2015. Adverse event following immunization surveillance systems for pregnant women and their infants: A systematic review. Pharmacoepidemiology and Drug Safety, 24, 361-367.  GUPTA, S., CAMPBELL, H., DOLAN GAYLE, P., KAPADIA SMITA, J., ANDREWS, N. & AMIRTHALINGAM, G. 2014. Vaccination in pregnancy to prevent pertussis in early infancy. Cochrane Database of Systematic Reviews (Protocol) |
| **Commentary/letters** |
| CHERRY, J. D. 2015. Tetanus-diphtheria-pertussis immunization in pregnant women and the prevention of pertussis in young infants. Clinical Infectious Diseases, 60, 338-40.  KHARBANDA, E. O., VAZQUEZ-BENITEZ, G., LIPKIND, H. S., KLEIN, N. P., CHEETHAM, T. C., NALEWAY, A., OMER, S. B., HAMBIDGE, S. J., LEE, G. M., JACKSON, M. L., MCCARTHY, N. L., DESTEFANO, F. &  NORDIN, J. D. 2015. Evaluation of the association of maternal pertussis vaccination with obstetric events and birth outcomes: Editorial comment. Obstetrical and Gynecological Survey, 70, 153-155.  MAERTENS, K., HOANG, T. H., CABORE, R. N. & LEURIDAN, E. 2015. Avidity of maternal pertussis antibodies after vaccination during pregnancy. Vaccine, 33, 5489.  MCINTYRE, P. B. & CLARK, T. A. 2014. Pertussis vaccine in pregnancy - First dose for every infant? The Lancet, 384, 1484-1486.  MILLAR, M. R. & SANZ, M. G. 2015. The administration of pertussis vaccine to pregnant women was associated with a small increased risk of chorioamnionitis, but not an increased risk of hypertensive disorders or preterm birth. Evidence Based Medicine, 20, 73.  MITCHELL, A. A., LOUIK, C., CHAMBERS, C., JONES, K. L. & SCHATZ, M. 2015. Immunization surveillance systems for pregnant women. Pharmacoepidemiology and Drug Safety, 24, 669.  SAFADI, M. A. 2015. Control of pertussis in infants: time has finally come? Expert Review of Vaccines, 14, 781-3.  SUKUMARAN, L., MCCARTHY, N. L., KHARBANDA, E. O., MCNEIL, M. M., NALEWAY, A. L., KLEIN, N. P., JACKSON, M. L., HAMBIDGE, S. J., LUGG, M. M., LI, R., WEINTRAUB, E. S., BEDNARCZYK, R. A., KING, J. P., DESTEFANO, F., ORENSTEIN, W. A. & OMER, S. B. 2016. Association of TDAP Vaccination with Acute Events and Adverse Birth Outcomes among Pregnant Women with Prior Tetanus-Containing Immunizations. Obstetrical and Gynecological Survey, 71, 1-2.  VILAJELIU, A., GARCIA-BASTEIRO, A. L. & BAYAS, J. M. 2015. Protecting newborns against pertussis: the value of vaccinating during pregnancy. Expert Review of Vaccines, 14, 1051-3.  ZHU, Y., VAN BOEMMEL-WEGMANN, S. & ALBOGAMI, Y. 2016. Repeat Tdap Vaccination and Adverse Birth Outcomes. JAMA, 315, 1285-6. |
| **Conference abstract** |
| JUDY, A., SINGH, A., LEE, H., GASKARI, S., BRODZINSKY, L., VIK, J., DRUZIN, M., EL-SAYED, Y. & AZIZ, N. 2015. TDaP vaccination safety in pregnancy: A comparison of neonatal and obstetric outcomes among women receiving antepartum and postpartum vaccination. American Journal of Obstetrics and Gynecology, 1), S300-S301.  MORGAN, J., BAGGARI, S., MCINTIRE, D. & SHEFFIELD, J. 2015. Use of a best-practice alert to impact prenatal tetanus toxoid, reduced diphtheria toxoid and acellular pertussis vaccination rates. American Journal of Obstetrics and Gynecology, 1), S97-S98.  STURGEON, T. E. & HAQUE, L. 2013. Maternal vaccination for Pertussis prevention-a review of pre and post-vaccination programme data. BJOG: An International Journal of Obstetrics and Gynaecology, 120, 494.  VURAL, M., ERENER ERCAN, T., SONMEZ, C., ERGINOZ, E., TORUNOGLU, M. A. & PERK, Y. 2015. Seropre valance of pertussis antibodies in maternal and cord blood of preterm and term infants. Journal of Perinatal Medicine. Conference: 12th World Congress of Perinatal Medicine, 43. |
