# Supplementary material for: Efficacy and safety of pertussis vaccination for pregnant women – a systematic review of randomised controlled trials and observational studies
Source: BMC Pregnancy Childbirth. 2017 Nov 22;17:390. doi: 10.1186/s12884-017-1559-2 (PMC5700667; doi:10.1186/s12884-017-1559-2)
Supplement: Supplementary file 3 — Risk of bias assessment. The risk of bias assessment for all included studies using the Cochrane collaboration risk of bias tools (DOCX 16 kb) [file 12884_2017_1559_MOESM3_ESM.docx]

Additional File 3: Risk of bias assessment

| **RCTs** | *Sequence generation* | *Allocation concealment* | | *Blinding* | | | *Incomplete*  *outcome* | | *Selective outcome reporting* | | *Other sources of bias* |
| --- | --- | --- | --- | --- | --- | --- | --- | --- | --- | --- | --- |
| Munoz 2014 [31] | ? | ? | | ✓^a^ | | ?^b^ | ✓ | | ? | | ? |
| Hoang 2016 [30] | ? | ? | | ✓^a^ | | ?^b^ | ? | | ✓ | | ? |
| **Cohort Studies** | *Selection of cohorts* | *Assessment of exposure (antenatal pertussis vaccination)* | | *Matching or adjustment* *for prognostic factors* | | | *Assessment of prognostic factors* | | *Assessment of outcomes* | | *Follow up* |
| Abu Raya 2011 [26] | ? | ? | | ? | | | ✓ | | ✓ | | ✓ |
| Abu Raya 2011 [27] | ? | ? | | ? | | | ? | | ✓ | | × |
| De Schutter 2015 [28] | ? | ✓ | | ? | | | ? | | ✓ | | ? |
| Donegan 2014 [32] | ? | ✓ | | × | | | ? | | ✓ | | ✓ |
| Gall 2011 [5] | ? | ? | | × | | | - | | ✓ | | ? |
| Hardy-Fairbanks 2013 [33] | ? | ? | | × | | | ? | | ✓ | | × |
| Healy 2013 [34] | ✓ | × | | ? | | | ? | | ✓ | | ✓ |
| Kharbanda 2014 [24] | ✓ | ✓ | | ? | | | ? | | ? | | ? |
| Kharbanda 2016 [25] | ✓ | ✓ | | ? | | | ? | | ? | | ? |
| Ladhani 2015 [35] | ? | ✓ | | × | | | - | | ✓ | | ? |
| Maertens 2016 [29] | ? | ✓ | | ? | | | ? | | ✓ | | ? |
| Shakib 2013 [36] | ✓ | ✓ | | × | | | - | | ✓ | | ? |
| **Case-control study** | *Assessment of exposure (antenatal maternal pertussis vaccination* | | *Assessment of primary outcome*  *(pertussis diagnosis)* | | *Case selection* | | | *Control selection* | | *Matching or adjustment for prognostic factors* | |
| Dabrera 2015 [37] | ✓ | | ✓ | | ? | | | ? | | ? | |

✓Low risk of bias; × High risk of bias; ? Unclear risk of bias; - Not applicable

1. Objectively measured outcomes
2. Subjectively measured outcomes
