# Supplementary material for: Efficacy and safety of pertussis vaccination for pregnant women – a systematic review of randomised controlled trials and observational studies
Source: BMC Pregnancy Childbirth. 2017 Nov 22;17:390. doi: 10.1186/s12884-017-1559-2 (PMC5700667; doi:10.1186/s12884-017-1559-2)
Supplement: Supplementary file 4 — Efficacy and safety of pertussis vaccination for pregnant women – A systematic review of randomised controlled trials and observational studies (Protocol). A protocol developed prior to the review. (PDF 783 kb) [file 12884_2017_1559_MOESM4_ESM.pdf]

Efficacy and safety of pertussis vaccination for pregnant women – A systematic review of randomised controlled trials and observational studies (Protocol)

Marie Furuta

16 Feb 2015

Table of Contents

1. BACKGROUND **4**

2. AIM AND OBJECTIVES **6**

3. METHODS **6**

3.1 Study Design 6

3.2 Inclusion and Exclusion Criteria 6

3.2.1 Types of participants *6*

3.2.2 Types of interventions or exposures *6*

3.2.3 Comparators *6*

3.2.4 Types of outcome measures *7*

3.2.5 Types of studies *9*

3.3 Search Methods for Identification of Studies 10

3.3.1 Search strategy *10*

3.4 Data Collection and Analysis 10

3.4.1 Selection of studies *10*

3.4.2 Data extraction and management *10*

3.4.3 Assessment of risk of bias in included studies *11*

3.4.4 Measures of treatment effect *12*

3.4.5 Multiplicity and unit of analysis issues *12*

3.4.6 Dealing with missing data *13*

3.4.7 Assessment of reporting biases *14*

3.4.8 Data analysis *14*

3.4.9 Subgroup analysis *15*

3.4.10 Sensitivity analysis *15*

Reference 16

APPENDICES **18**

Appendix 1 Search strategies 18

Cochrane Central Register of Controlled Trials (CENTRAL) search strategy *18*

Medline (OvidSp) search strategy *19*

Embase search strategy *20*

Open Gray (all years) *21*

APPENDIX 2 DATA EXTRACTION FORM 22

APPENDIX 3 RISK OF BIAS ASSESSMENT FOR RCTS/QUASI-RCTS 32

APPENDIX 4 RISK OF BIAS ASSESSMENT FOR OBSERVATIONAL STUDIES 34

1. Background

Worldwide, pertussis remains one of the most common causes of hospitalisation and death in infants (Amirthalingam, 2013). Despite a widely applied infant vaccination programme against pertussis that has a high coverage rate, many industrialised countries have recently been experiencing a national outbreak of this preventable disease. Improved case ascertainment has meant that more cases are being recognised in adolescents and adults. But waning immunity following vaccination (which typically occurs 4 to 12 years after the last booster dose or episode of illness) and decreasing natural boosting of immunity also play a role in the increased incidence of pertussis (Amirthalingam, 2013, Leuridan et al., 2011). Pertussis in young adults is a serious public health issue because it can be a source of infection for newborns and very young infants who have not yet been vaccinated, and newborn infants are the population at the highest risk of serious health complications, such as pneumonia, seizures, brain damage and death (Leuridan et al., 2011).

A number of potential strategies to control pertussis in infants have been proposed. One that was introduced quite recently involves vaccinating women in the third trimester of pregnancy. In 2011, in an effort to reduce the incidence of pertussis in infants, the United States (US) became the first country to recommend that health-care personnel administer a dose of tetanus toxoid, reduced diphtheria toxoid, and acellular pertussis vaccine (Tdap) to pregnant women between 27 and 36 weeks gestation who had not previously received Tdap in adulthood (CDC, 2013b). The American College of Obstetricians and Gynecologists (ACOG) and the American College of Nurse-Midwives (ACNM) both support the recommendation to use Tdap in the the third trimester for pregnant women (CDC, 2013a).

In 2012, the Department of Health for England also began offering a pertussis vaccination for all women in late pregnancy to help protect their babies against pertussis when the United Kingdom experienced a nationwide outbreak of pertussis from which 14 babies under 3 months died (Oxford vaccine group, 2015). It was the highest pertussis mortality rate in the country since 1982, when there were also 14 recorded pertussis deaths (Billingsley, 2012). Since the programme started, the national coverage of pertussis immunisation in pregnancy has increased from 44% in the first month of the programme to around 60% in 2014 (Public Health England, 2015). Based on an observational study, Amirthalingam et al. (2014) evaluated the effectiveness of the vaccination programme and concluded that ‘maternal

immunisation with an acellular pertussis-containing vaccine can provide about 90% protection against infant disease' (p.6).

How the intervention might work

Vaccinating women in the third trimester of pregnancy is likely to protect infants from a pertussis infection in at least two ways. First, it may increase the transfer of beneficial maternal antibodies to the fetus. Earlier studies have shown that cord blood from newborns whose mothers received Tdap during or before pregnancy had higher concentrations of pertussis antibodies compared to cord blood from the children of unvaccinated mothers, suggesting the existence of efficient transplacental transfer of pertussis antibodies (Gall et al., 2011, Leuridan et al., 2011). Second, vaccination during pregnancy is likely to prevent maternal infection at the time of delivery and therefore minimize the infant's potential exposure to pertussis (Amirthalingam, 2013).

Why it is important to do this review

Given a newborn's immune system is not able to create antibodies until he/she is 2 months old, maternal immunisation is considered the only option currently available to protect infants against pertussis infection from birth until the first infant vaccinations are given at the age of 2 months (Amirthalingam, 2013, CDC, 2015). Vaccination in pregnancy is now recommended for women in some countries, including the UK, the US, New Zealand, and Belgium (Amirthalingam, 2014). Although evaluations of the maternal immunisation programme in the UK have shown some favourable results, little is known regarding the quality of the evidence. Further robust evidence from a systematic review of existing studies is required to evaluate the efficacy of maternal antenatal vaccinations to protect newborn infants from pertussis. Also, evaluating adverse effects of maternal vaccinations is crucial to addressing a clinical and policy question: Should maternal pertussis vaccinations be supported and become routine for all pregnant women, or should immunisation be reserved as a countermeasure during outbreaks (McIntyre and Clark, 2014)?

2. Aim and Objectives

This study aims to examine the efficacy and safety of the pertussis vaccinations given to pregnant women to protect infants from pertussis infection.

Primary Objective

- To examine the efficacy of maternal pertussis vaccinations in reducing the incidence of infant pertussis compared to placebo or no vaccination.

Secondary Objectives

- To examine the efficacy of maternal pertussis vaccinations on infants' and mothers' immune responses.
- To examine the efficacy of maternal pertussis vaccinations on the prevention of severe pertussis infections (as measured in hospital admissions, severe complications, and mortality attributed to pertussis).
- To examine the safety of maternal pertussis vaccinations for mothers and infants (as measured by adverse vaccine-related outcomes in response to vaccination and obstetric or perinatal complications).

3. Methods

3.1 Study Design

A systematic review of experimental studies (e.g., randomised controlled trials [RCTs] and quasi-RCTs) and observational studies.

3.2 Inclusion and Exclusion Criteria

3.2.1 Types of participants

Mothers and their babies in any settings.

3.2.2 Types of interventions or exposures

A pertussis vaccine or pertussis-containing vaccine (e.g., Tdap, dTap-IPV) during pregnancy.

3.2.3 Comparators

No vaccination or placebo vaccination during pregnancy.

3.2.4 Types of outcome measures

Primary Outcome

- Incidence of pertussis (either laboratory-confirmed or clinically diagnosed pertussis) in infants up to 12 months of age.

Although any diagnostic tests will have some limitations (Box 1), clinical symptoms and signs demonstrate poor accuracy for diagnosing pertussis (Shojaei et al., 2014). The impact of including clinically diagnosed pertussis in the outcome will be explored by undertaking sensitivity analyses, if necessary (see sensitivity analyses section below).

Box 1 Cases of pertussis

Clinical case definition
Pertussis is defined as a cough illness lasting at least 2 weeks with one of the following symptoms: paroxysms of coughing, inspiratory 'whoop', or post-tussive vomiting without other apparent cause (as reported by a health-care professional; Faulkner et al., 2011). Atypical pertussis symptoms are common in young infants (Castagnini and Munoz, 2010, Eidlitz-Markus et al., 2007). Therefore, illnesses caused by other respiratory microbes are often misdiagnosed as pertussis (Shojaei et al., 2014, Cosnes-Lambe et al., 2008, Walsh et al., 2011, Korppi and Hiltunen, 2007). For an accurate diagnosis of pertussis, laboratory confirmation of a clinical pertussis illness is required (Cherry et al., 2005, Shojaei et al., 2014).

Laboratory diagnosis
Culture
Isolation of *Bordetella pertussis (B. pertussis)* by bacterial culture is the only 100% specific method (i.e., no false positives) and is therefore considered the gold standard for laboratory case confirmation (WHO, 2007). However, the sensitivity of the culture is poor, with generally less than a 60% success rate in identifying cases of pertussis infection. Diagnosis by culture is most successful with a nasopharyngeal (NP) swab or aspirate clinical specimen collected from patients with suspected pertussis during the catarrhal stage (the first 1 to 2 weeks of coughing), when viable (live) bacteria are still present in the nasopharynx (CDC, 2013a). After the first 2 weeks of coughing, however, the risk of false negatives (in which positive cases may be misclassified as negative) increases (Faulkner et al., 2011).

Polymerase chain reaction (PCR)
The polymerase chain reaction (PCR) is a molecular test used to detect DNA sequences of the *B. pertussis* bacterium. Unlike isolating by taking a bacterial culture, PCR can provide timely results and does not require viable bacteria in the specimen (Faulkner et al., 2011). Instead, it requires bacterial DNA; it is therefore recommended that PCR should be tested from an NP swab or aspirated clinical specimen taken at 0–3 weeks following the onset of coughing, when bacterial DNA is still present in the nasopharynx. After the fourth week of coughing, the amount of bacterial DNA reduces rapidly, which increases the risk of false negatives [20](CDC, 2011a). Although testing for PCR is more sensitive and less likely to produce false negatives when compared with taking cultures, specificity can be poor, with high rates of false positives. False positives can occur as a result of *B. pertussis* DNA contamination of clinical specimens. It has been reported that some pertussis vaccines, including Tdaps Daptacel® and Adacel®, contain PCR-detectable *B. pertussis* DNA. To avoid false positive results, only patients with pertussis-like symptoms (e.g., prolonged coughing with paroxysms and/or whooping or choking) should be tested for PCR (CDC, 2011a, CDC 2011b). In other words, an asymptomatic person with a positive PCR should not usually be considered a case of pertussis infection (CDPH, 2010).

Serology
Serological tests measure antibodies (e.g., serum immunoglobulin G [IgG] and IgA antibodies) against *B. pertussis* antigens (e.g., pertussis toxin [PT] and filamentous hemagglutinin [FHA]). Commercially, several different serologic tests are available (e.g., enzyme-linked immunosorbent assays [ELISAs]). These serologic assays can be useful for diagnosing pertussis in later phases of the disease, when both cultures and PCR tests are unlikely to be positive (CDC, 2011a). Serologic tests, however, measure antibodies resulting from either natural infections or vaccinations, so high-titre antibody responses simply mean that the person has been exposed to pertussis by infection or by vaccination. Because pertussis vaccines consist of various components of the *B. pertussis* bacterium (Poolman and Hallander, 2007), use of such serologic assays cannot differentiate infection from vaccine response without the presence of clinical symptoms of pertussis (CDC, 2011b).

Secondary Outcomes

Efficacy

- Incidence of pertussis in infants up to 2 months of age (prior to the first dose of a pertussis vaccine), from 2 to 6 months of age (after at least the first dose of a pertussis vaccine), and from 6 to 12 months of age (likely having been administered 3 primary doses of a pertussis vaccine).
- Hospitalisation, severe complications, and/or mortality attributed to pertussis in infants up to 2 months of age, from 2 to 6 months of age, and from 6 to 12 months of age.
- Mothers' and infants' immune responses (e.g., IgG and IgA antibodies to pertussis toxin [PT], filamentous hemagglutinin [FHA], Pertactin [PRN]; Fimbriae 2 and 3 [FIM 2/3]) in maternal and infant blood after intervention, at delivery, and 2 months postpartum and up to 12 months of age.

Safety

- Incidence of any local reactions (e.g., pain, redness, or swelling) at the site of injection.
- Incidence of maternal systemic reactions (e.g., drowsiness, fretfulness, decreased appetite, fever, febrile seizures, or anaphylaxis) following injection.
- Incidence of adverse obstetric or perinatal outcomes (e.g., chorioamnionitis, hypertensive disorders, preterm births, small-for-gestational-age births, stillbirth or neonatal death).

3.2.5 Types of studies

We will include RCTs in which individual mothers are randomly assigned to vaccination or no vaccination and quasi-RCTs in which individual mothers are assigned to vaccination or no vaccination using some rule (eg. odd or even date of birth). This method will ensure that differences in outcomes—such as incidence of pertussis in babies born to mothers who received the pertussis vaccine as compared to those born to unvaccinated mothers—can be examined. We will also include observational studies because the administration of maternal pertussis vaccinations is a recent policy recommendation and has been applied in limited settings. Thus, restricting this review to only experimental studies might severely limit the amount of data available to evaluate the efficacy and safety of the vaccination.

3.3 Search Methods for Identification of Studies

3.3.1 Search strategy

Electronic Searches

Relevant studies will be identified in the following databases:

- Cochrane Central Register of Controlled Trials (CENTRAL)
- Ovid MEDLINE
- Embase
- OpenGREY.

The search strategy is shown in Appendix 1.

Searching Other Resources

Additional searches will be undertaken by a hand search of the reference lists of included studies.

3.4 Data Collection and Analysis

3.4.1 Selection of studies

Citations retrieved from the searches will be imported into the reference management software package EndNote X7. After removing duplicates, the list of titles, abstracts, and descriptors/MeSH terms will be screened for relevance. Each study will be coded as 'retrieve' (eligible or potentially eligible/unclear) or 'do not retrieve'. Studies coded as 'retrieve' will be further assessed with the full texts to determine whether the studies met the inclusion criteria or to record reasons for excluding the ineligible studies. The process of the study selection will be recorded in detail so as to complete a PRISMA flow diagram and 'characteristics of excluded studies' table.

3.4.2 Data extraction and management

The following information will be extracted onto the data extraction form that was designed specifically for this review (Appendix 2):

- Study design (e.g., experimental or observational studies, methods of sample selection/recruitment)
- Inclusion/exclusion criteria
- Participants' demographics and clinical characteristics (e.g., countries, parity)

- Incidence/prevalence of pertussis in the population being studied
- Intervention (e.g., setting of intervention and the type and timing of the vaccination)
- Comparison (no vaccination or placebo vaccination)
- Outcomes (primary and secondary measures; methods used to measure outcomes; length of follow-up, including age cut-offs the study used to identify pertussis cases)
- Results (reported statistics and number of participants lost or excluded at each stage of the trial)

Data included in analyses will be extracted onto an Excel spreadsheet before transporting them onto Review Manager (RevMan) 5.3 (or STATA 13) software.

3.4.3 Assessment of risk of bias in included studies

RCT

The risk of bias of the included RCTs will be assessed using the approach recommended in the *Cochrane Handbook for Systematic Review of Interventions* (Higgins, 2011). The Cochrane Collaboration's tool for assessing risk of bias, which is mainly aimed at parallel groups and individually randomised trials, addresses six specific domains: (1) sequence allocation for randomization, (2) allocation concealment, (3) blinding of personnel and assessors, (4) incomplete outcome data, (5) selective reporting, and (6) any other notable risks of bias. For each item, one of the following three judgements will be made when insufficient information is reported to permit judgment: low risk of bias (plausible bias that is unlikely to alter the results seriously), high risk of bias (plausible bias that seriously weakens confidence in the results), or unclear risk of bias (plausible bias that raises some doubt about the results; see Appendix 3 for further details on risk of bias assessment).

Observational Studies

For risk of bias in observational studies, this review adapted the Cochrane Collaboration risk of bias tool for nonrandomised studies (Cochrane Bias Methods Group, 2014). See Appendix 4 for further details on risk-of-bias assessment for observational studies.

Summary Assessments of Risk of Bias

The overall quality of the evidence for each outcome will be assessed using the The Grades of Recommendation, Assessment, Development and Evaluation Working Group (GRADE)

approach (Higgins and Green, 2011). We will provide information regarding the process for judging the quality of evidence and the magnitude of the effect of the pertussis vaccination.

3.4.4 Measures of treatment effect

Dichotomous Data

For dichotomous outcomes such as the incidence of pertussis, the Mantel-Haenszel method for computing the pooled risk ratio (RR) with 95% confidence intervals (CI) will be used.[1] RR, rather than odds ratio (OR), will be used in meta-analyses because OR often leads to overestimation of the benefits and harms of the intervention when the results are applied in clinical practice (Higgins and Green, 2011, Section 9.4.4.4). However, ORs will be calculated for case-control studies. (In a case-control study, the entire population at risk cannot be defined; thus, it is not possible to calculate RR.)

Continuous Data

The weighted mean difference (WMD) and 95% CI will be calculated where all outcomes will be measured in the same way, while the standardised mean difference (SMD) will be calculated if different scales were used.

3.4.5 Multiplicity and unit of analysis issues

Multiple Outcomes or Repeated Measures

To avoid incorrect estimates of the variance for the summary effect, the same patients should not appear more than once in each meta-analysis (Borenstein et al., 2009). If a study reported data on more than one outcome or time point, analyses would have been conducted separately for each set of outcomes/time points, such as short term (< 2 months of age—prior to the first dose of a pertussis vaccine), medium term (from 2 to 6 months of age—in which infants are likely to get at least the first dose of a pertussis vaccine), and long term (from 6 months of age forward, at which point infants have likely been given three primary doses of a pertussis vaccine but will need additional booster doses).

[1] If a study had no events either in the intervention or control group, RevMan software calculated the risk ratio (RR) automatically by adding a fixed value (0.5) to each cell of the 2 x 2 table.

Multiple Comparisons

In the trials with multiple interventions (or controls), participants in the control group (or the intervention group) often contribute information to more than one effect size, which, again, leads to a wrong estimate of the pertussis variance. If this review identifies a trial that involved multiple comparisons, combining data to create a single pair-wise comparison will be considered if interventions (or controls) are sufficiently similar as recommended by the Cochrane Handbook for Systematic Reviews (Higgins and Green, 2011, Section 16.5). Alternatively, data from the arms of the trial that fit closest to the review objective will be used with a detailed description of why particular groups are selected in the table of "Characteristics of included studies."

Crossover Trials

Crossover trials are not suitable for evaluating interventions with a lasting effect due to the issue of carry-over, which occurs if an effect of maternal vaccination in a first pregnancy is carried over into the subsequent pregnancy, resulting in systematic differences in participants when they enter the second phase of the study. Therefore, when a study adopted a crossover design, only outcome data from the first randomisation period will be included.

Cluster-randomised Trials

In cluster-randomised trials (where groups of individuals are randomised to different groups rather than individuals), individuals' data can no longer be assumed to be independent of one another (Higgins and Green, 2011). Because the purpose of this review is to examine the efficacy and the safety at the individual level, only studies that reported patient-level data will be included.

3.4.6 Dealing with missing data

The degree to which missing data becomes problematic depends on the pattern (i.e., data are systematically missing related to a treatment) and amount of missing values (Little and Rubin, 2002). To deal with missing data, this review plans to conduct an intention-to-treat (ITT) analysis for dichotomous outcomes. This would involve sensitivity analysis by imputing outcomes for the missing participants with the best-case scenario (i.e., assuming that the infant whose data are missing turns out to be pertussis negative) and with the worst-case scenario (i.e., assuming that the infant whose data are missing turns out to be pertussis positive) and then comparing the results of the two analyses. The sensitivity analysis will also

compare the result of ITT with imputations from available case analyses (i.e., analyse data with participants whose outcomes are known, excluding any participants whose outcomes are missing from the denominator for each outcome in each trial). Where data are missing for mean or standard deviations, we will calculate them from standard errors (SEs), confidence intervals, or t-values using the RevMan calculator.

3.4.7 Assessment of reporting biases

When sufficient studies are available (n = 10 or more), this review will create funnel plots to investigate the possibility of publication bias (Higgins and Green, 2011).

3.4.8 Data analysis

Data Synthesis

Random effects meta-analyses will be performed to produce the average effect size of the intervention across studies. Random effects meta-analyses (a conservative option) is more appropriate than a fixed-effect model (which assumes that there is one true effect) because population and setting of trials are likely slightly different; therefore, the effects are likely slightly different. However, in the situation where there are only a few studies (two to four studies), it is more appropriate to perform a fixed-effect analysis, because random effects meta-analyses cannot accurately estimate the width of the distribution of intervention effects (Higgins and Green, 2011, Kontopantelis et al., 2013). The results obtained from the two methods (random effects and fixed-effect models) will then be compared to seek potential bias and heterogeneity. This review will include both randomised controlled trials and observational studies. Data from observational studies will be analysed separately from trial data.

Heterogeneity

Clinical heterogeneity (variability in the interventions and control, participants and settings, and outcomes) and methodological heterogeneity (variability in study design and risk of bias) will be assessed within each comparison. If comparable studies are not available with significant clinical heterogeneity, extracted data will be synthesised into a narrative summary. Where meta-analyses are performed, tests of statistical heterogeneity will be further carried out using I^2 and Chi^2 statistics (Higgins and Green, 2011) as well as visual inspection of the forest plots. Study results will be reported separately if there is significant heterogeneity between findings of different studies.

3.4.9 Subgroup analysis

If there are sufficient data, we will conduct further subgroup analyses by considering sources of possible clinical heterogeneity:

- Study Setting

  Rationale: there may be heterogeneity in vaccine response between different settings (e.g., high-, middle-, and low-income countries) in which the general health statuses of mothers and infants as well as the countries' health-care systems differ.
- Timing of Vaccination

  Rationale: Pertussis vaccinations can be administered at any time during pregnancy, but current recommendations by both the CDC Advisory Committee on Immunization Practices (ACIP) and the ACOG regarding optimal timing for administering the vaccination is between 27 and 36 weeks gestation.
- Type of Vaccination

  Rationale: there can be heterogeneity in vaccine response across different types of vaccinations (whole-cell vaccine vs. acellular vaccine).

3.4.10 Sensitivity analysis

We will conduct a sensitivity analysis to assess the effects of quality of trial methodology by comparing the results of meta-analyses with and without trials that are judged to have a high risk of bias (e.g., a bias in the domains of accuracy of outcome measures [using clinically diagnosed pertussis vs. laboratory confirmed], random sequence generation, allocation concealment, blinding of outcome assessment, and/or incomplete outcome). A sensitivity analysis will also be conducted to examine potential bias caused by missing data by comparing results from different methods of dealing with missing data (e.g., available case analysis and ITT analysis using imputation of outcomes, assuming that all missing participants had positive outcomes or that all missing participants had negative outcomes).

Reference

AMIRTHALINGAM, G. 2013. Strategies to control pertussis in infants. *Archives of Disease in Childhood,* 98**,** 552-5.

AMIRTHALINGAM, G., ANDREWS, N., CAMPBELL, H., RIBEIRO, S., KARA, E., DONEGAN, K., FRY, N. K., MILLER, E. & RAMSAY, M. 2014. Effectiveness of maternal pertussis vaccination in England: an observational study. *The Lancet*.

BILLINGSLEY, M. 2012. Pregnant women in UK are offered whooping cough vaccine to protect newborns. *BMJ,* 345**,** e6594.

BORENSTEIN, M., HEDGES, L., HIGGINS, J. & ROTHSTEIN, H. R. 2009. *Introduction to meta-analysis*, John Wiley & Sons, Ltd.

CALIFORNIA DEPARTMENT OF PUBLIC HEALTH (CDPH). 2010. Pertussis: Laboratory Testing. Available: http://www.cdph.ca.gov/programs/immunize/Documents/CDPH_Pertussis%20laboratory%20testing_March2010.pdf [Accessed 3rd January 2015].

CASTAGNINI, L. A. & MUNOZ, F. M. 2010. Clinical characteristics and outcomes of neonatal pertussis: a comparative study. *J Pediatr,* 156**,** 498-500.

CENTERS FOR DISEASE CONTROL AND PREVENTION (CDC). 2011a. Best practices for health care professionals on the use of polymerase chain reaction (PCR) for diagnosing pertussis. Available: http://www.cdc.gov/pertussis/clinical/diagnostic-testing/diagnosis-pcr-bestpractices.html [Accessed 3rd January 2015].

CENTERS FOR DISEASE CONTROL AND PREVENTION (CDC) 2011b. Epidemiology and prevention of vaccine-preventable diseases. *In:* ATKINSON, W., WOLFE, S. & HAMBORSKY, J. (eds.) 12th ed. Washington DC: Public Health Foundation.

CENTERS FOR DISEASE CONTROL AND PREVENTION (CDC). 2013a. *Pertussis (Whooping Cough): diagnosis confirmation* [Online]. Available: http://www.cdc.gov/pertussis/clinical/diagnostic-testing/diagnosis-confirmation.html [Accessed 3rd January 2015].

CENTERS FOR DISEASE CONTROL AND PREVENTION (CDC) 2013b. Updated recommendations for use of tetanus toxoid, reduced diphtheria toxoid, and acellular pertussis vaccine (Tdap) in pregnant women--Advisory Committee on Immunization Practices (ACIP), 2012. *MMWR - Morbidity & Mortality Weekly Report,* 62**,** 131-5.

CENTERS FOR DISEASE CONTROL AND PREVENTION (CDC). 2015. *Pregnancy and Whooping Cough* [Online]. Available: http://www.cdc.gov/pertussis/pregnant/mom/vaccinate-baby.html [Accessed Feb 2 2016].

CHERRY, J. D., GRIMPREL, E., GUISO, N., HEININGER, U. & MERTSOLA, J. 2005. Defining pertussis epidemiology: clinical, microbiologic and serologic perspectives. *Pediatr Infect Dis J,* 24**,** S25-34.

COCHRANE BIAS METHODS GROUP. 2014. *Research Projects: Cochrane Risk of Bias tool* [Online]. Available: https://bmg.cochrane.org/research-projectscochrane-risk-bias-tool [Accessed 15 January 2014].

COSNES-LAMBE, C., RAYMOND, J., CHALUMEAU, M., PONS-CATALANO, C., MOULIN, F., DE SUREMAIN, N., REGLIER-POUPET, H., LEBON, P., POYART, C. & GENDREL, D. 2008. Pertussis and respiratory syncytial virus infections. *Eur J Pediatr,* 167**,** 1017-9.

EIDLITZ-MARKUS, T., MIMOUNI, M. & ZEHARIA, A. 2007. Pertussis symptoms in adolescents and children versus infants: the influence of vaccination and age. *Clin Pediatr (Phila),* 46**,** 718-23.

FAULKNER, A., SKOFF, T., MARTIN, S., CASSIDAY, P., TONDELLA, M., LIANG, J. & EJIGIRI, O. 2011. Chapter 10: Pertussis. *In:* CENTERS FOR DISEASE CONTROL AND PREVENTION (CDC) (ed.) *Manual for the surveillance of vaccine-preventable diseases.* Atlanta, GA: CDC.

GALL, S. A., MYERS, J. & PICHICHERO, M. 2011. Maternal immunization with tetanus-diphtheria-pertussis vaccine: effect on maternal and neonatal serum antibody levels. *American Journal of Obstetrics & Gynecology,* 204**,** 334.e1-5.

HIGGINS, J. & GREEN, S. P. 2011. *Cochrane Handbook for Systematic Reviews of Interventions,* Oxford, Wiley-Blackwell.

KONTOPANTELIS, E., SPRINGATE, D. A. & REEVES, D. 2013. A Re-Analysis of the Cochrane Library Data: The Dangers of Unobserved Heterogeneity in Meta-Analyses. *Plos One,* 8.

KORPPI, M. & HILTUNEN, J. 2007. Pertussis is common in nonvaccinated infants hospitalized for respiratory syncytial virus infection. *Pediatr Infect Dis J,* 26**,** 316-8.

LEURIDAN, E., HENS, N., PEETERS, N., DE WITTE, L., VAN DER MEEREN, O. & VAN DAMME, P. 2011. Effect of a prepregnancy pertussis booster dose on maternal antibody titers in young infants. *Pediatric Infectious Disease Journal,* 30**,** 608-10.

LITTLE, R. J. A. & RUBIN, D. B. 2002. *Statistical analysis with missing data,* Chichester, Wiley.

MCINTYRE, P. B. & CLARK, T. A. 2014. Pertussis vaccine in pregnancy—first dose for every infant? *The Lancet*.

OXFORD VACCINE GROUP. 2015. *Vaccines in Pregnancy: Pertussis (whooping cough)* [Online]. Available: http://www.ovg.ox.ac.uk/pertussis-vaccine-in-pregnancy [Accessed].

POOLMAN, J. T. & HALLANDER, H. O. 2007. Acellular pertussis vaccines and the role of pertactin and fimbriae. *Expert Rev Vaccines,* 6**,** 47-56.

PUBLIC HEALTH ENGLAND. 2015. *Pertussis Vaccination Programme for Pregnant Women: vaccine coverage estimates in England, April to August 2014* [Online]. Available: https://www.gov.uk/government/publications/pertussis-immunisation-in-pregnancy-vaccine-coverage-estimates-in-england-october-2013-to-march-2014/pertussis-vaccination-programme-for-pregnant-women-vaccine-coverage-estimates-in-england-april-to-august-2014 [Accessed 16 Feb 2015].

SHOJAEI, J., SAFFAR, M., HASHEMI, A., GHORBANI, G., REZAI, M. & SHAHMOHAMMADI, S. 2014. Clinical and laboratory features of pertussis in hospitalized infants with confirmed versus probable pertussis cases. *Ann Med Health Sci Res,* 4**,** 910-4.

WALSH, P. F., KIMMEL, L., FEOLA, M., TRAN, T., LIM, C., DE SALVIA, L., PUSAVAT, J., MICHAELSON, S., NGUYEN, T. A., EMERY, K., MORDECHAI, E. & ADELSON, M. E. 2011. Prevalence of Bordetella pertussis and Bordetella parapertussis in infants presenting to the emergency department with bronchiolitis. *J Emerg Med,* 40**,** 256-61.

WORLD HEALTH ORGANIZATION (WHO). 2007. Laboratory manual for the diagnosis of whooping cough caused by Bordetella pertussis/Bordetella parapertussis. Available: http://whqlibdoc.who.int/hq/2004/WHO_IVB_04.14_eng.pdf [Accessed 3rd January 2015].

Appendices

Appendix 1 Search strategies

Cochrane Central Register of Controlled Trials (CENTRAL) search strategy

#1	neonat*
#2	MeSH descriptor: [Postpartum Period] explode all trees
#3	MeSH descriptor: [Infant, Newborn] explode all trees
#4	MeSH descriptor: [Infant] this term only
#5	maternal
#6	matern*
#7	perinatal
#8	Pregnan*
#9	MeSH descriptor: [Mothers] this term only
#10	(#1 or #2 or #3 or #4 or #5 or #6 or #7 or #8 or #9)
#11	MeSH descriptor: [Pertussis Vaccine] explode all trees
#12	MeSH descriptor: [Vaccination] explode all trees
#13	MeSH descriptor: [Immunization] this term only
#14	immunisation
#15	MeSH descriptor: [Immunization Programs] this term only
#16	MeSH descriptor: [Diphtheria-Tetanus-Pertussis Vaccine] this term only
#17	MeSH descriptor: [Diphtheria-Tetanus-acellular Pertussis Vaccines] this term only
#18	Whole-cell pertussis vaccine
#19	Tdap
#20	dTap-IPV
#21	(#11 or #12 or #13 or #14 or #15 or #16 or #17 or #18 or #19 or #20)
#22	pertussis
#23	MeSH descriptor: [Whooping Cough] this term only
#24	MeSH descriptor: [Antibodies, Bacterial] explode all trees
#25	anti-pertussis antibodies
#26	IgG
#27	MeSH descriptor: [Immunoglobulin G] explode all trees
#28	IgA
#29	MeSH descriptor: [Immunoglobulin A] explode all trees
#30	MeSH descriptor: [Antigen-Antibody Reactions] explode all trees
#31	MeSH descriptor: [Drug-Related Side Effects and Adverse Reactions] explode all trees
#32	MeSH descriptor: [Pre-Eclampsia] explode all trees
#33	MeSH descriptor: [Eclampsia] explode all trees
#34	MeSH descriptor: [Hypertension, Pregnancy-Induced] explode all trees
#35	MeSH descriptor: [Pregnancy Outcome] explode all trees
#36	MeSH descriptor: [Pregnancy Complications] explode all trees
#37	MeSH descriptor: [Puerperal Disorders] explode all trees
#38	MeSH descriptor: [Infant, Premature] explode all trees
#39	MeSH descriptor: [Infant, Small for Gestational Age] explode all trees
#40	(#22 or #23 or #24 or #25 or #26 or #27 or #28 or #29 or #30 or #31 or #32 or #33 or #34 or #35 or #36 or #37 or #38 or #39)
#41	(#10 and #21 and #40)

Medline (OvidSp) search strategy

1	maternal.mp. [mp=title, abstract, original title, name of substance word, subject heading word, keyword heading word, protocol supplementary concept word, rare disease supplementary concept word, unique identifier]
2	matern*.mp.
3	perinatal.mp.
4	Pregnan*.mp.
5	mother.mp.
6	exp Mothers/
7	1 or 2 or 3 or 4 or 5 or 6
8	exp Pertussis Vaccine/
9	vaccination*.mp.
10	exp Vaccination/
11	exp Vaccines/
12	exp Vaccines, Inactivated/
13	exp Vaccines, Acellular/
14	Immuni?ation*.mp.
15	immunisation.mp.
16	Immunization/
17	exp Immunization Programs/
18	exp Diphtheria-Tetanus-Pertussis Vaccine/
19	exp Diphtheria-Tetanus-acellular Pertussis Vaccines/
20	Whole-cell pertussis vaccine.mp.
21	Tdap.mp.
22	dTap-IPV.mp.
23	8 or 9 or 10 or 11 or 12 or 13 or 14 or 15 or 16 or 17 or 18 or 19 or 20 or 21 or 22
24	pertussis.mp. or exp Whooping Cough/
25	exp Antibodies, Bacterial/
26	anti-pertussis antibodies.mp.
27	IgG.mp.
28	exp Immunoglobulin G/
29	IgA.mp.
30	exp Immunoglobulin A/
31	exp Antigen-Antibody Reactions/
32	exp "Drug-Related Side Effects and Adverse Reactions"/
33	exp Pre-Eclampsia/
34	exp Eclampsia/
35	exp Hypertension, Pregnancy-Induced/
36	exp Pregnancy Outcome/
37	exp Pregnancy Complications/
38	exp Puerperal Disorders/
39	exp Infant, Premature/
40	exp Infant, Small for Gestational Age/
41	24 or 25 or 26 or 27 or 28 or 29 or 30 or 31 or 32 or 33 or 34 or 35 or 36 or 37 or 38 or 39 or 40
42	7 and 23 and 41

Embase search strategy

1	maternal.mp. [mp=title, abstract, subject headings, heading word, drug trade name, original title, device manufacturer, drug manufacturer, device trade name, keyword]
2	matern*.mp.
3	perinatal.mp.
4	exp pregnancy/ or exp pregnant woman/ or pregnant.mp.
5	exp expectant mother/ or exp mother/
6	1 or 2 or 3 or 4 or 5
7	exp diphtheria pertussis tetanus Haemophilus influenzae type b vaccine/ or exp pertussis vaccine/ or exp diphtheria pertussis poliomyelitis tetanus hepatitis B vaccine/ or exp diphtheria pertussis tetanus hepatitis B vaccine/ or exp diphtheria pertussis poliomyelitis tetanus Haemophilus influenzae type b vaccine/ or exp diphtheria pertussis tetanus vaccine/ or exp diphtheria pertussis poliomyelitis tetanus vaccine/ or exp diphtheria pertussis poliomyelitis tetanus Haemophilus influenzae type b hepatitis B vaccine/ or exp pertussis toxin/ or exp diphtheria pertussis tetanus Haemophilus influenzae type b hepatitis B vaccine/
8	exp acellular vaccine/ or exp bacterial vaccine/ or vaccine/
9	exp inactivated vaccine/
10	exp vaccination/
11	immunisation.mp. or exp immunization/
12	Tdap.mp.
13	dTap-IPV.mp.
14	7 or 8 or 9 or 10 or 11 or 12 or 13
15	whooping cough.mp. or exp pertussis/
16	exp antibody response/ or exp immune response/
17	exp bacterium antibody/
18	exp immunoglobulin/ or exp immunoglobulin G/ or exp "antibody and immunoglobulin structure, function and production"/ or exp immunoglobulin A antibody/ or exp "antibody and immunoglobulin production"/ or exp immunoglobulin G antibody/ or exp immunoglobulin G deficiency/ or exp immunoglobulin A/
19	IgA.mp.
20	IgG.mp.
21	exp adverse drug reaction/ or exp side effect/ or exp drug effect/
22	exp "eclampsia and preeclampsia"/ or exp eclampsia/
23	exp preeclampsia/
24	exp hypertension/
25	exp low birth weight/ or exp premature labor/
26	exp small for date infant/
27	exp prematurity/
28	15 or 16 or 17 or 18 or 19 or 20 or 21 or 22 or 23 or 24 or 25 or 26 or 27
29	6 and 14 and 28

Open Gray (all years)

- “Bordetella pertussis”
- “Bordetella pertussis” AND Vaccine
- “Bordetella pertussis” AND Vaccination
- “Whooping cough” AND Vaccine
- “Whooping cough” AND Vaccination

Appendix 2 Data extraction form

GENERAL

Inclusion: ☐ Yes ☐ No

Specify reasons for exclusion:

Date of data extraction:

Identification of the reviewer:

Study identification:

Author(s):

Article Title:

Source (Journal, Conference) Year / Volume / Pages / Country of Origin:

Institutional Affiliation (first author) and/or contact details:

Peer review (journal): ☐ Yes ☐ No

METHODS:

Data collection period:

I. Study design:

☐ Randomised controlled trial (RCT)[2]

☐ Controlled clinical trial (CCT)[3]

☐ Cohort study

☐ Case-control study

☐ Cross-sectional study

II. Inclusion/exclusion criteria:

Inclusion:

Exclusion:

III. Participants' demographic and clinical characteristics:

Country:

Ethnicity:

Age:

Parity:

---

[2] Randomised controlled trial (RCT) is a trial in which the participants (or other units) were randomly assigned to different study arms

[3] Controlled clinical trial (CCT) may be a trial in which participants (or other units) were:
a) participants assigned to different study arms using a quasi-random allocation method (e.g. alternation, date of birth, patient identifier) or; b) participants assigned to different study arms using a process of random or quasi-random allocation.

IV. Incidence/prevalence of pertussis in the population being studied:

V. Characteristics of the intervention:

Setting of intervention:

☐ Hospital/clinic/GPs

☐ Other (describe)

Type:

☐ Tdap

☐ Other (describe) : ____________

Timing of the vaccination:

VI. Comparison:

☐ Placebo

☐ No vaccination

☐ Other (describe) : ____________

VII. Outcome measures

Primary outcome of the original study:

Methods used to measure primary outcome:

Secondary outcome(s) of the original study:

Methods used to measure secondary outcome(s):

What was measured at baseline?

What was measured after the intervention?

Who carried out the measurement?

Length of follow-up:

Measurement of outcomes of interests in this review

1. Incidence of pertussis ☐ Yes (measured) ☐ No (not measured)

   If yes,

   Data source

   ☐ Laboratory diagnosis

   ☐ Culture

   ☐ PCR

   ☐ Serology

   ☐ Other (describe): _____________

   ☐ Clinical diagnosis

   Length of follow-up

   ☐ <12 months of age

   ☐ < 2 months of age

   ☐ 2 to 6 months of age

   ☐ 6 to 12 months of age

2. Hospitalisation, severe complications, incidence of mortality attributable to pertussis

   <u>Hospitalisation</u> ☐ Yes ☐ No

   If yes,

   Data source

   ☐ Clinical record

   ☐ Self-report

   ☐ Other (describe): _____________

   Length of follow-up

   ☐ <12 months of age

   ☐ < 2 months of age

   ☐ 2 to 6 months of age

   ☐ 6 to 12 months of age

<u>Severe complications</u> ☐ Yes ☐ No

If yes,

Type(s) of complications

☐ Pneumothorax

☐ Pneumonia

☐ Encephalopathy (a diffuse disorder of the brain)

☐ Seizures

☐ Other (describe): ____________

Data source

☐ Clinical record

☐ Self-report

☐ Other (describe): ____________

Length of follow-up

☐ <12 months of age

☐ < 2 months of age

☐ 2 to 6 months of age

☐ 6 to 12 months of age

<u>Incidence of mortality attributable to pertussis</u> ☐ Yes ☐ No

If yes,

Data source

☐ Clinical record

☐ Other (describe): ____________

Length of follow-up

☐ <12 months of age

☐ < 2 months of age

☐ 2 to 6 months of age

☐ 6 to 12 months of age

3. Anti-pertussis antibodies titre (IgG and IgA) in mothers ☐ Yes ☐ No

If yes,

Type(s) of anti-pertussis antibodies measured

☐ IgG

☐ IgA

Length of follow-up

☐ 4 weeks after vaccination

☐ At delivery

☐ 2 months postpartum

☐ Other (describe): ____________

4. Anti-pertussis antibodies titre (IgG and IgA) in infants ☐ Yes ☐ No

If yes,

Type(s) of anti-pertussis antibodies measured

☐ IgG

☐ IgA

Length of follow-up

☐ <12 months of age

- ☐ < 2 months of age
- ☐ 2 to 6 months of age
- ☐ 6 to 12 months of age

5. Incidence of local reactions at the site of injection ☐ Yes ☐ No

If yes,

Type(s) of local reactions

☐ Pain

☐ Redness

☐ Swelling

☐ Other (describe): ____________

Data source

☐ Clinical record

☐ Self-report

☐ Other (describe): ____________

6. Incidence of maternal systemic reactions following injections ☐ Yes ☐ No

If yes,

Type(s) of systemic reactions

☐ Drowsiness

☐ Fretfulness

☐ Decreased appetite

☐ Fever

☐ Other (describe): ____________

Data source

☐ Clinical record

☐ Self-report

☐ Other (describe): ____________

7. Incidence of adverse obstetric outcomes ☐ Yes ☐ No

If yes,

Type(s) of adverse outcomes

☐ Chorioamnionitis

☐ Hypertensive disorders (gestational hypertension, preeclampsia or eclampsia)

☐ Other (describe): ____________

Data source

☐ Clinical record

☐ Self-report

☐ Other (describe): ____________

8. Incidence of preterm and small-for-gestational-age births ☐ Yes ☐ No

If yes,

☐ Preterm

☐ Small-for-gestational-age

Data source

☐ Clinical record

☐ Self-report

☐ Other (describe): ____________

VIII. Data extraction

	Entire study		Intervention or exposures		Control	
	N	%	N	%	N	%
Number of participants identified						
Number of eligible participants						
Number of participants included						
Number of participants randomised						
Excluded after randomisation						
Lost to follow-up						
Withdrawals						
Final number of participants evaluable						
Clear description of withdrawals and exclusions	☐ Yes<br>☐ No		☐ Yes<br>☐ No		☐ Yes<br>☐ No	

NR: Not Reported

IX. Analysis

Dichotomous outcomes						
Outcomes	Intervention or exposure group (n)			Control group (n)		
	N	OR	RR *(CI 95%)*	N	OR	RR *(CI 95%)*
pertussis in infants						

Continuous outcomes						
Outcomes	Unit of measurement	Intervention or exposure group		Control group		Details if outcome only described in text
		n	Mean (SD)	n	Mean (SD)	

Power calculation reported? ☐ Yes ☐ No

Analysis by Intention to Treat (ITT): ☐ Yes ☐ No

Subgroup analyses: ☐ Yes ☐ No

If Yes, specify the groups:

The Cochrane Collaboration’s tool for assessing risk of bias for RCT<br><br>**Outcome:**		
**Bias Domain**	**Review authors’ judgment**	**Description**
Sequence generation	☐ low risk<br>☐ high risk<br>☐ unclear	
Allocation concealment	☐ low risk<br>☐ high risk<br>☐ unclear	
Blinding of participants, personnel and outcome assessors	☐ low risk<br>☐ high risk<br>☐ unclear	
Incomplete outcome data	☐ low risk<br>☐ high risk<br>☐ unclear	
Selective outcome reporting	☐ low risk<br>☐ high risk<br>☐ unclear	
Other sources of bias	☐ low risk<br>☐ high risk<br>☐ unclear	

The Cochrane Collaboration's tool for assessing risk of bias for cohort study<br><br>Outcome:		
**Bias Domain**	**Review authors' judgment**	**Description**
Was selection of vaccinated and unvaccinated cohorts drawn from the same population?	☐ low risk<br>☐ high risk<br>☐ unclear	
Can we be confident in the assessment of exposure (antenatal maternal pertussis vaccination)?	☐ low risk<br>☐ high risk<br>☐ unclear	
Did the study match exposed and unexposed for all variables that are associated with the outcome of interest or did the statistical analysis adjust for these prognostic variables?	☐ low risk<br>☐ high risk<br>☐ unclear	
Can we be confident in the assessment of the presence or absence of prognostic factors?	☐ low risk<br>☐ high risk<br>☐ unclear	
Can we be confident in the assessment of outcome?	☐ low risk<br>☐ high risk<br>☐ unclear	
Was the follow up of cohorts adequate?	☐ low risk<br>☐ high risk<br>☐ unclear	

The Cochrane Collaboration's tool for assessing risk of bias for case-control study<br><br>**Outcome:**		
**Bias Domain**	**Review authors' judgment**	**Description**
Can we be confident in the assessment of exposure?	☐ low risk<br>☐ high risk<br>☐ unclear	
Can we be confident that the outcome of interest (i.e. pertussis in infants, either laboratory diagnosed or clinically diagnosed) was assessed for both cases and controls?	☐ low risk<br>☐ high risk<br>☐ unclear	
Were the cases (those who were exposed and developed the outcome of interest) properly selected?	☐ not applicable<br>If applicable,<br>☐ low risk<br>☐ high risk<br>☐ unclear	
Were the controls (those who were exposed and did not develop the outcome of interest) properly selected?	☐ low risk<br>☐ high risk<br>☐ unclear	
Were cases and controls matched according to important prognostic variables or was statistical adjustment carried out for those variables?	☐ low risk<br>☐ high risk<br>☐ unclear	

Appendix 3 Risk of bias assessment for RCTs/quasi-RCTs

Sequence generation

The strategy used for sequence generation will be examined to judge the risk of possible selection bias as being:

- low risk (if an adequate process of sequence generation was used in which each participant had an equal chance of being randomised to a group—e.g., random number table, computer random number generator);
- high risk (if inadequate process of sequence generation was used—e.g., alternation, date of birth, hospital or clinic number); or
- unclear risk

Allocation concealment

The method used to conceal the allocation sequence will be examined to assess selection bias and judge the risk of it as being

- low risk (if adequate strategies were used for achieving allocation concealment—e.g., telephone or central randomization, pre-numbered or coded serial identical containers);
- high risk (if allocation was foreseen in advance of, or during, recruitment—e.g., alternation, date of birth); or
- unclear risk

Blinding of personnel and assessors

Assessments of risk of bias resulting from lack of blinding (performance bias, possible detection bias) will be made separately for different outcomes.

- low risk (e.g., blinding of participants and key study personnel—administers of vaccines, assessors of outcomes and data analysts—ensured, and unlikely that the blinding could have been broken)
- high risk (e.g., blinding of participants and key study personnel not blinded)
- unclear risk

Incomplete outcome data

By examining the amount and distribution of missing data, reasons for outcomes being missing, and how missing data were handled in the analysis, the risk of attrition bias (systematic difference between group caused by withdrawals, dropouts, and/or protocol deviations) will be assessed as follows:

- low risk (e.g., low proportion of outcome data missing; the proportion of and reasons for missing outcome data both reported and balanced across groups, ITT analysis conducted for dealing with missing data)
- high risk (e.g., high proportion of outcome data missing; the proportion of or reasons for missing outcome data imbalanced across groups; "as-treated" (or "per-protocol") analysis[4] performed with substantial difference between the intervention received and that assigned at randomisation)
- unclear risk

Selective reporting

Reporting bias (systematic differences between reported and unreported findings) will be assessed for each study by comparing the outcomes reported in the results with the protocols. Risk of bias will be assessed as being

- low risk (e.g., all prespecified outcomes and all expected outcomes of interest adequately reported),
- high risk (e.g., not all prespecified outcomes reported, outcomes not prespecified reported), or
- unclear risk

Other notable risks of bias

Any other possible sources of bias that are not addressed in the domains mentioned above will be assessed, including issues such as adherence to study protocol, and differences between the intervention and control groups at baseline.

[4] Only participants who received the intended intervention in accordance with the protocol are included in the analysis (Higgins et al. 2008)

Appendix 4 Risk of bias assessment for observational studies

Cohort Studies

(Adapted from Cochrane Bias Methods Group, 2014)

1. **Was selection of vaccinated and unvaccinated cohorts drawn from the same population?**
    - low risk (both vaccinated and unvaccinated cohorts drawn from the same population)
    - high risk (vaccinated and unvaccinated cohorts drawn from different populations)
    - unclear risk

2. **Can we be confident in the assessment of exposure (antenatal maternal pertussis vaccination)?**
    - low risk (data on vaccination status obtained from medical records)
    - high risk (women's self-report such as being told by a physician that they had a vaccination)
    - unclear risk (uncertain how exposure information obtained)

3. **Can we be confident that the outcome of interest was not present at start of study?**
    This question is not relevant to the primary outcome (incidence of pertussis in infants) and other secondary outcomes in infants because the exposure is antenatal maternal pertussis vaccination, thus, the outcome of interest will never be present at the start of the study.

4. **Did the study match exposed and unexposed for all variables that are associated with the outcome of interest or did the statistical analysis adjust for these prognostic variables?**
    - low risk (comprehensive matching or adjustment for selection of pre-specified relevant variables)
    - high risk (no or little matching/adjustment or relevant variables used for matching/adjustment not pre-specified. Statements of no differences between groups or that differences were not statistically significant are not sufficient for establishing comparability)
    - unclear risk

5. **Can we be confident in the assessment of the presence or absence of prognostic factors?**
    - low risk (medical records, database documented)
    - high risk (self-report)
    - unclear risk

6. **Can we be confident in the assessment of outcome?**
    - low risk (eg. laboratory diagnosis of pertussis using culture or PCR with pertussis-like symptoms)
    - high risk (eg. pertussis diagnosed with clinical symptoms and signs)
    - unclear risk

7. **Was the follow up of cohorts adequate?**
    - low risk (reasons for missing outcome data unlikely to be related to true outcome; missing outcome data balanced in numbers across intervention groups, with similar reasons for missing data across groups; missing data have been imputed using appropriate methods).
    - high risk (reason for missing outcome data likely to be related to true outcome, with either imbalance in numbers or reasons for missing data across intervention groups)
    - unclear risk

<u>Case Control Studies</u>

(Adapted from Cochrane Bias Methods Group, 2014)

1. **Can we be confident in the assessment of exposure?**
    - low risk (secure record such as written vaccination records)
    - high risk (women's self-report such as being told by a physician that they had a vaccination)
    - unclear risk (uncertain how exposure information obtained)

2. **Can we be confident that the outcome of interest (i.e. pertussis in infants, either laboratory diagnosed or clinically diagnosed) was assessed for both cases and controls?**
    - low risk (cases and controls undergo valid and reliable diagnostic procedures— i.e., laboratory diagnosis of pertussis using culture or PCR with pertussis-like symptoms)
    - high risk (eg. cases and controls were identified by clinical symptoms and signs without laboratory tests)
    - unclear risk

3. **Were the cases (those who were exposed and developed the outcome of interest) properly selected?**
    - low risk (all eligible cases are enrolled in a defined catchment area over a defined period of time during which diagnostic procedures would be unlikely to have changed, or a random sample of those cases)
    - high risk (eligible cases in a defined catchment area over a defined period of time during which diagnostic procedures would be likely to have changed, or a random sample of those cases)
    - unclear risk

4. **Were the controls (those who were exposed and did not develop the outcome of interest) properly selected?**
    - low risk (the cohort of mothers whose infants did not develop pertussis was selected from the same population as the cohort of mothers whose infants developed pertussis)
    - high risk (the cohort of mothers whose infants did not develop pertussis was not selected from the same population as the cohort of mothers whose infants developed pertussis)
    - unclear risk

5. **Were cases and controls matched according to important prognostic variables or was statistical adjustment carried out for those variables?**
    - low risk (comprehensive matching or adjustment for selection of pre-specified relevant variables)
    - high risk (no or little matching/adjustment or relevant variables used for matching/adjustment not pre-specified. Statements of no differences between groups or that differences were not statistically significant are not sufficient for establishing comparability)
    - unclear risk
